# Casein kinase 1.2 over expression restores stress resistance to *Leishmania donovani* HSP23 null mutants

**DOI:** 10.1038/s41598-020-72724-x

**Published:** 2020-09-29

**Authors:** Constanze Kröber-Boncardo, Stephan Lorenzen, Christine Brinker, Joachim Clos

**Affiliations:** 1 Leishmania Group, Bernhard Nocht Institute for Tropical Medicine, Bernhard Nocht St 74, 20359 Hamburg, Germany; 2 Department of Epidemiology, Bernhard Nocht Institute for Tropical Medicine, Hamburg, Germany

**Keywords:** Post-translational modifications, Parasitology, Chromosomes, DNA recombination, Post-translational modifications, Cell biology, Microbiology, Molecular biology, Systems biology, Infectious diseases, Parasitic infection

## Abstract

*Leishmania donovani* is a trypanosomatidic parasite and causes the lethal kala-azar fever, a neglected tropical disease. The Trypanosomatida are devoid of transcriptional gene regulation and rely on gene copy number variations and translational control for their adaption to changing conditions. To survive at mammalian tissue temperatures, *L. donovani* relies on the small heat shock protein HSP23, the loss of which renders the parasites stress sensitive and impairs their proliferation. Here, we analysed a spontaneous escape mutant with wild type-like in vitro growth. Further selection of this escape strains resulted in a complete reversion of the phenotype. Whole genome sequencing revealed a correlation between stress tolerance and the massive amplification of a six-gene cluster on chromosome 35, with further analysis showing over expression of the casein kinase 1.2 gene as responsible. In vitro phosphorylation experiments established both HSP23 and the related P23 co-chaperone as substrates and modulators of casein kinase 1.2, providing evidence for another crucial link between chaperones and signal transduction protein kinases in this early branching eukaryote.

## Introduction

Protozoan parasites of the genus *Leishmania* cause upwards of one million known infections of humans annually^1^, with an unknown number of non-symptomatic carriers. The resulting immune pathologies, summarily known as leishmaniasis, are among the most important of the neglected tropical diseases (NTDs) as defined by the WHO^2^. In addition, the leishmaniae share many important molecular features with the other Trypanosomatida, namely *Trypanosoma cruzi* and *Trypanosoma brucei*, the causative agents of the NTDs Chagas Disease and Human African Trypanosomiasis (HAT), respectively. The most severe form of leishmaniasis, visceral leishmaniasis, is caused by the closely related *L. donovani* and *L. infantum* and results in persistent fever, enlargement of spleen and liver, a depletion of antigen-presenting cells, anaemia and a concomitantly increased susceptibility to secondary infections. Without effective chemotherapy, this form of the disease is lethal. There is no effective vaccine or prophylaxis, and the chemotherapeutics against leishmaniasis are fraught with drawbacks such as toxic side effects, teratogenicity, high costs, inconsistent efficacy and/or existing or developing resistance^3,4^.

From the biological viewpoint, the most striking feature of trypanosomatidic protozoa is their complete lack of canonical, gene-specific regulation of transcription. *Leishmania* chromosomes consist of long, polycistronic transcription units of functionally non-related genes^5–7^ that are transcribed into polycistronic precursor RNAs. Precursor RNAs are then processed via trans-splicing coupled to polyadenylation to yield mature, monocistronic mRNAs^8,9^. This is reflected by the lack of genes for transcription regulator protein orthologs in the parasites' genomes. While differential RNA degradation and/or processing is likely responsible for fluctuating mRNA levels^9–11^, short-term regulation of gene expression likely relies on regulated translation^12–15^.

Another genetic feature of *Leishmania* is a natural, constitutive aneuploidy^16–18^, but also the ability to generate and maintain quantitative genetic diversity through intrachromosomal or episomal amplification of genes or gene clusters. Inverted and non-inverted repeat sequences of the SIDER family frame and facilitate such gene amplificates^19,20^, causing gene copy number variations^21^ in the parasite populations from which the best adapted variants may be selected under adverse conditions such as drug pressure^22–25^. This may also happen after artificial genetic changes in the laboratory. Spontaneous reversal of phenotypes was described for LPG2^−/−^- and HSP100^−/−^-null mutants of *L. major*^26,27^. Such phenotype reversals can even be mimicked by functional cloning using cosmid libraries of *Leishmania* genomic DNA^28,29^.

*Leishmania* parasites reside as flagellated, elongated promastigotes attached to the midgut walls of infected phlebotomine sandflies from where they are transmitted to mammals including humans. Taken up by host antigen-presenting cells, they undergo morphological stage conversion to non-flagellated, non-motile, ovoid amastigotes that reside within the phagosomes mainly of macrophages^30,31^. The sandfly stage is also characterised by a rapid, logarithmic growth while the amastigote has a reduced proliferation rate and can even enter quiescence^32–34^*.* Another consequence of the transmission from arthropod to mammalian hosts is the dual trigger of mammalian tissue temperatures and the acidified milieu inside macrophage phagosomes. Indeed, in vitro exposure of axenically cultivated promastigotes to both increased temperature (33–37° C, depending on the *Leishmania* species) and slightly acidic growth medium (pH 5–5.5) can trigger the morphological and metabolic shift to an amastigote-like cell^35–37^*.*

At the same time, the temperature shift also induces a classic cellular stress response, with increased synthesis of several heat shock protein families, including HSP100/ClpB^38,39^, HSP90 and HSP70^12^, a 60 kDa chaperonin (CPN60.2)^40^ and its likely co-chaperone CPN10^41^.

Another chaperone, the small heat shock protein 23 (HSP23) also shows a temperature-dependent expression, and HSP23 null mutants (HSP23^−/−^) indeed suffer a severe viability loss at mammalian tissue temperature (37 °C), impaired chemical stress tolerance and a reduced growth at permissive temperatures^42^. HSP23 belongs to the rather diverse small HSP family that are characterised primarily by the so-called α-crystallin domain, but has a unique overall domain structure compared with other small HSPs and is more closely related to the p23 co-chaperone of *Leishmania* which modulates the accessibility of the ATPase domain of the major chaperone HSP90 for HSP90 inhibitors^43^.

In this paper, we describe the spontaneous reversal of the temperature-sensitive (TS) phenotype of *L. donovani* HSP23^−/−^ null mutants under in vitro selection at permissive and non-permissive temperatures. The escape variants display wild-type level temperature tolerance and in vitro proliferation rates and chemoresistance, due to a massive amplification of the casein kinase 1.2 (CK1.2) gene. We further show that HSP23, but also P23, are substrates of CK1.2, establishing yet another link^44,45^ between protein kinase-mediated signalling and the chaperone machinery of *Leishmania*.

## Results

### Emergence of an escape variant of HSP23^−/−^

**Figure 1:**
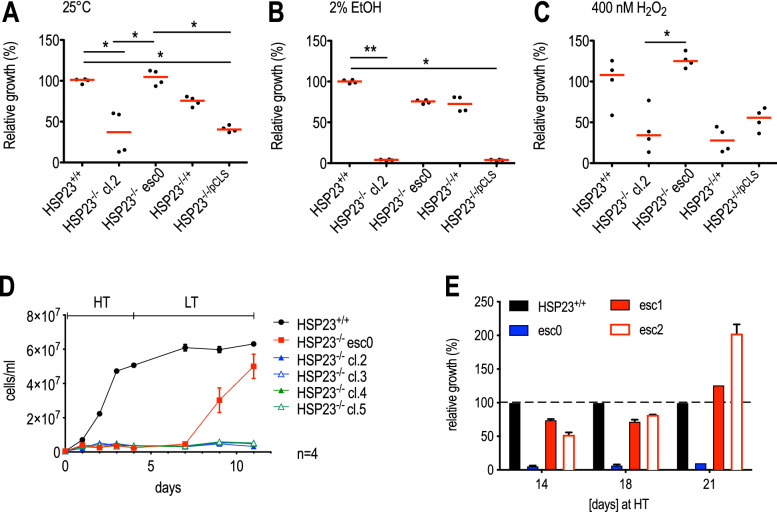
Phenotype comparison of HSP23^−/−^ and HSP23^−/−^ escape mutants under different culture conditions. 1✕10^6^ cells were seeded into 7 ml of supplemented M199 + medium. Cell density on day 4 post seeding was then normalised to wild-type (HSP23^+/+^) cell density (set at 100%). Parasites were grown at LT (25 °C) (**A**), 2% EtOH at LT (**B**) or 400 nM H_2_O_2_ at LT (**C**). Differences were tested for significance using the Kruskal–Wallis test: *p < 0.05, **p < 0.01 (n = 4) (**D**) Heat selection of HSP23^−/−^ esc0: 5✕10^5^ cells/ml were seeded into 10 ml of M199 + medium and incubated for 4 days at HT (37 °C). The cultures were transferred back to LT and grown to mid-log phase for seven days. (**E**) Growth analysis of two heat selected (**D**) HSP23^−/−^ mutant populations at HT, the non selected HSP23^−/−^ esc0 population and HSP23^+/+^ wild type cells (set as 100%). Cells were diluted twice weekly to 5✕10^5^ cells/ml. Error bars represent the standard error of the mean (SEM).

It was previously shown that the loss of HSP23 renders *Leishmania donovani* promastigotes incapable of surviving at mammalian tissue temperatures (hereafter referred to as high temperature (HT)) and more sensitive towards several stressors such as ethanol and acidic pH. Moreover, in vitro proliferation at 25 °C (hereafter referred to as low temperature (LT)) was also affected^42^. After approximately 25 in vitro passage cycles at LT, however, an escape population emerged with wild type-like growth phenotype which we designated HSP23^−/−^ esc0. We also generated new HSP23^−/−^ null mutants, following the same recombination strategy as used for the parental strain of esc0^42^. The clone 2 was tested and still shows the original, growth impaired phenotype (Fig. 1A). As expected, HSP23^−/−^ cl.2 growth can be restored by episomal expression of a HSP23 transgene (HSP23^−/−/+^), but not by the expression vector alone (HSP23^−/−/pCLS^).

Very similar results were obtained under challenge with 2% ethanol. Here, growth of HSP23^−/−^ cl.2 and HSP23^−/−/pCLS^ was almost completely suppressed. By contrast, HSP23^−/−^ esc0 and HSP23^−/−/+^ showed no significant impairment relative to wild type (HSP23^+/+^) (Fig. 1B).

We also tested the oxidative stress sensitivity of wild type (HSP23^+/+^) and mutant strains by monitoring in vitro growth under H_2_O_2_ challenge (400 nM) (Fig. 1C). Again, HSP23^−/−^ esc0 was indistinguishable from wild type, while HSP23^−/−^ cl.2 and HSP23^−/−/pCLS^ were significantly affected, similar to the growth defects under standard culture conditions (25 °C, panel A). Surprisingly, the add-back strain, HSP23^−/−/+^, did not show restored growth under H_2_O_2_ resistance either, indicating a difference between add-back and escape strain.

Earlier work^42^ suggested that exposure of HSP23^−/−^ to HT was lethal to the mutant. To assess the survival of a panel of HSP23^−/−^ clones at elevated temperatures, we exposed wild type and null mutant clones to HT for 4 days, monitoring cell density daily (Fig. 1D). In keeping with earlier experiments, only wild type (HSP23^+/+^) showed growth at HT. Shifting back the cultures to LT, all HSP23^−/−^ clones except for HSP23^−/−^ esc0 showed no outgrowth, indicating that the 4-day exposure to HT had been indeed lethal to HSP23^−/−^ clones 2 and 3. The HSP23^−/−^ esc0 alone showed outgrowth starting at 2 days after the temperature downshift, indicating that a substantial number of promastigotes had survived the preselection.

Based on this assumption, we compared two of the outgrowth populations with the original HSP23^−/−^ esc0 strain and wild type for their ability to sustain growth at HT over a period of 21 days. The result of this experiment is shown in Fig. 1E; growth of the mutants is shown in [%] of wild type growth. While the original, not preselected strain (esc0) shows only marginal growth, the two populations (esc1, esc2) coming from the preselection shown in Fig. 1D show increasing vitality over the course of the experiment, reaching a relative growth exceeding that of wild type cells in the last days. Obviously, the preselection at HT followed by unimpeded growth at LT led to the spontaneous emergence of two fully temperature tolerant strains while the parent strain esc0 is not capable of growth at 37 °C. This indicates that esc0, while showing increased survival at 37 °C compared with the other HSP23-/- clones, required a recovery period to fully express temperature tolerance.

### Genetic Adaptions of HSP23^−/−^ Mutants

In lieu of transcriptional control *Leishmania* gene expression is often modulated by gene dosage through chromosomal aneuploidies^18,46^, gene copy number variations^20,47^ or selection of circular and linear amplicons^19,48,49^. In particular, such genomic alterations are frequently found in leishmaniae after challenge with stressful conditions such as drug pressure or environmental changes^50,51^.

To identify the genomic alterations that compensate for the lack of HSP23 at permissive and non-permissive temperatures, we performed whole genome sequencing of *L. donovani* 1S wild type selected at LT (n = 2) and HT (n = 2), HSP23^−/−^ clone 2 (n = 1) and clone 3 (n = 1) selected at LT, HSP23^−/−^ esc0 selected at LT (n = 2) and HSP23^−/−^ esc1 (n = 1) and esc2 (n = 1) selected at HT. The sequence read data are available at https://www.ncbi.nlm.nih.gov/sra/PRJNA633969.

Firstly, we verified the accuracy of the *HSP23* gene replacements for HSP23^−/−^ cl.2, HSP23^−/−^cl.3, and HSP23^−/−^ esc mutants (Supplementary Figs. S1, S2), finding the homologous recombination to have worked entirely as expected.

Using NGS sequence read coverage data, we analysed the aneuploidy patterns (Fig. S3, Table S2). Karyotype differences between HSP23^+/+^ and HSP23^−/−^ clones and escape variants were observed. Full or mosaic aneuploidy was found for chromosomes 8 and 35 in HSP23^−/−^ esc0, cl.2 and cl.3 at LT. Chromosomes 12 and 23 show a somy reduction in all HSP23^−/−^ escape variants while chromosome 14 shows mosaic trisomy in three of four HSP23^−/−^ escape samples.

**Figure 2:**
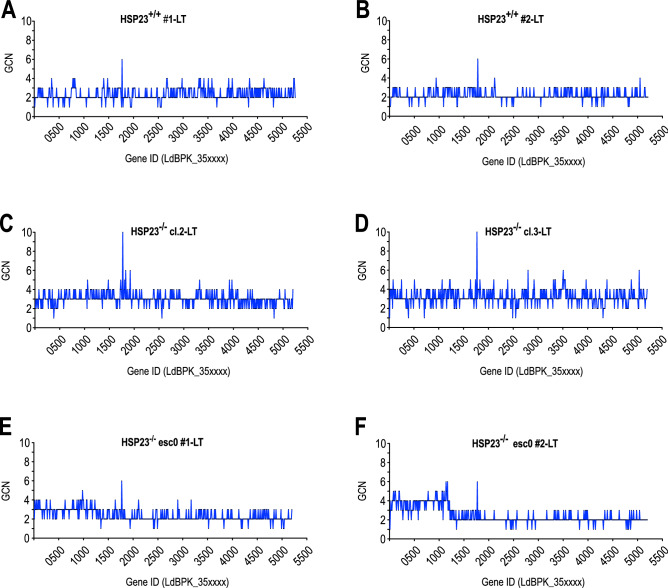
Strain-specific gene copy number variations on chromosome 35 at permissive temperatures. Normalised copy number for each gene (#1-#530) on chromosome 35 is shown for (**A**, **B**) HSP23^+/+^, (**C**, **D**) HSP23^−/−^, and (**E**, **F**) HSP23^−/−^ esc0. All strains were selected and grown at 25 °C (LT).

We next performed a genome-wide analysis of individual gene copy number variations for all strains subjected to whole genome sequencing. For the most part, changes of CNVs correlated with the observed somy changes (Table S2). However, the read depth analysis for chromosome 35 revealed striking discrepancies. As observed before, the HSP23^−/−^ clones 2 and 3 that were maintained at LT are trisomic for chromosome 35 which is reflected in an overall 50% increase of gene copy numbers compared with the wild type samples (Fig. 2 A–D). For both samples of HSP23^−/−^ esc0 maintained at LT we had observed a slight mosaic somy for chromosome 35 at 2.26 and 2.36 (Table S1). Gene copy number analysis, however, showed a selective amplification of a 0.55-megabase region at the 5′ end of chromosome 35 from nucleotide positions ~ 4500 bp to 554,500 bp and ~ 4500 bp to ~ 557,500 bp for sample 1 and sample 2, respectively. The observed amplifications encompass 124 and 126 genes, respectively, and result in 1.5- to twofold increase of gene copy numbers in these regions (Fig. 2E,F).

**Figure 3:**
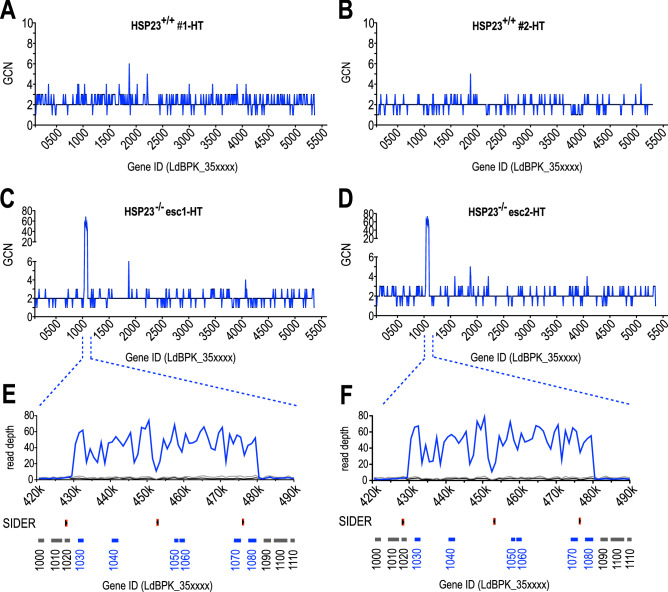
Strain-specific gene copy number variations on chromosome 35 at non-permissive temperatures. Normalised copy numbers for indicated genes (LdBPK_35xxxx) on chromosome 35 is shown for (**A**, **B**) HSP23^+/+^-HT, (**C**, **D**) HSP23^−/−^ esc1/2-HT, (**E**, **F**) enlargement of the amplified region on chromosome 35 between positions 420,000 and 490,000. Sequencing read coverage is shown for HSP23^+/+^ (LT, black), HSP23^−/−^ esc1/2 (HT, blue) and HSP23^−/−^ cl. 2/3 (LT, grey). Open reading frames and corresponding gene IDs are indicated in grey for non-amplified or blue for amplified CDSs; positions of SIDER2 repeat elements are indicated in red. LT:25 °C, HT:37 °C.

Analysis of the HSP23^−/−^ esc1 and -esc2 strains, both survivors of a 4-day HT challenge and able to grow at HT (Fig. 1D,E), displayed a more specific and extreme amplification. Compared with wild type cells selected at HT (Fig. 3A,B), esc1 and esc2 both show a 43- to 73-fold gene copy number increase for a cluster of six genes on chromosome 35 (Fig. 3C,D). This 49-kb region (position 430,500–479,500) is delimited by SIDER2 elements^19^ and codes for casein kinase 1.2 (CK1.2) (LdBPK_351030), two CCCH-type zinc finger proteins (LdBPK_351040 and LdBPK_351060), a cupin domain-containing protein (LdBPK_351050), an undefined putative protein kinase (LdBPK_351070) and a hypothetical protein (LdBPK_351080) (Fig. 3E,F).

**Figure 4:**
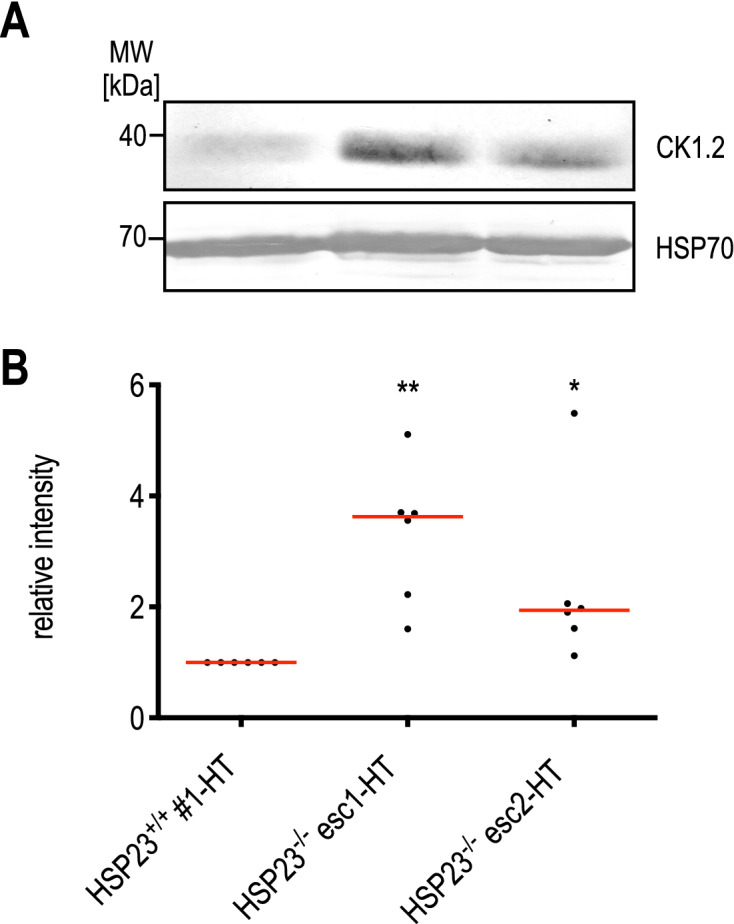
Abundance of CK1.2 in HSP23^−/−^ esc1/2 mutants. (**A**) Western blot analysis of CK1.2 in HSP23^ + / +^ and HSP23^−/−^ esc1/2 using a rabbit anti-CK1.2 antibody (SY3535) (1:250). A chicken anti-HSP70 (1:5000) antibody was used as a loading control. (**B**) CK1.2 protein level was quantified using ImageJ Fiji Software (Version 2.0.0) and first normalised to Coomassie loading control and then to the HSP23 ^+ / +^ (37 °C) sample. Statistical testing was carried out by Kruskal–Wallis test; *p < 0.05, **p < 0.01; (n = 6).

It has been previously demonstrated that karyotypic variation is associated with modulation of transcript abundance^23^. Thus, we questioned whether the observed amplification of the six-gene cluster on chromosome 35 in heat-resistant HSP23^−/−^ esc1/2 also changes RNA and protein abundance. Indeed, qRT-PCR analysis confirmed that amplification of the gene cluster in HSP23^−/−^ esc1/2 mutants results in a 10- to 41-fold increase of mRNA for the co-amplified genes (Table 1). With an anti-CK1.2 (LdBPK_351030) antibody available^52^, we performed Western blot analysis. Indeed, we observed a 2- to threefold up regulation of CK1.2 protein levels in the HSP23^−/−^ esc1/2 mutants, confirming the correlation between DNA copy numbers, RNA levels and protein abundance (Fig. 4A,B).

**Table 1:** Gene copy numbers (GCNs) and corresponding RNA levels of amplified candidates genes on chromosome 35 (LdBPK_351030-351080).

Gene ID: LdBPK	351030		351040		351050		351060		351070		351080	
	*GCN*	*RNA*	*GCN*	*RNA*	*GCN*	*RNA*	*GCN*	*RNA*	*GCN*	*RNA*	*GCN*	*RNA*
HSP23^+/+^ #1-LT	3	1.028 ± 0.06	2	1.050 ± 0.06	3	1.029 ± 0.04	3	0.988 ± 0.03	2	0.989 ± 0.01	2	0.998 ± 0.01
HSP23^−/−^ esc0 #1 -LT	4	1.445 ± 0.05	3	1.908 ± 0.07	4	1.798 ± 0.16	3	1.505 ± 0.19	5	1.64 ± 0.17	4	2.253 ± 0.17
HSP23^+/+^ #1-HT	2	0.915 ± 0.05	3	2.487 ± 0.32	3	1.120 ± 0.11	2	0.787 ± 0.11	2	0.953 ± 0.06	2	0.922 ± 0.15
HSP23^−/−^ esc1-HT	56	15.55 ± 1.6	53	35.60 ± 1.71	68	29.53 ± 3.42	48	10.60 ± 0.86	55	28.03 ± 2.55	43	40.72 ± 1.42
HSP23^−/−^ esc2-HT	63	13.11 ± 0.45	58	31.45 ± 1.28	73	30.95 ± 4.26	54	9.822 ± 1.05	62	26.44 ± 1.99	48	40.39 ± 6.05

RNA values are mean ± standard errors of the means (SEM). qRT-PCR data were obtained from three biological and two technical replicates each.

### Functional Cloning of Stress Tolerance Marker

**Figure 5:**
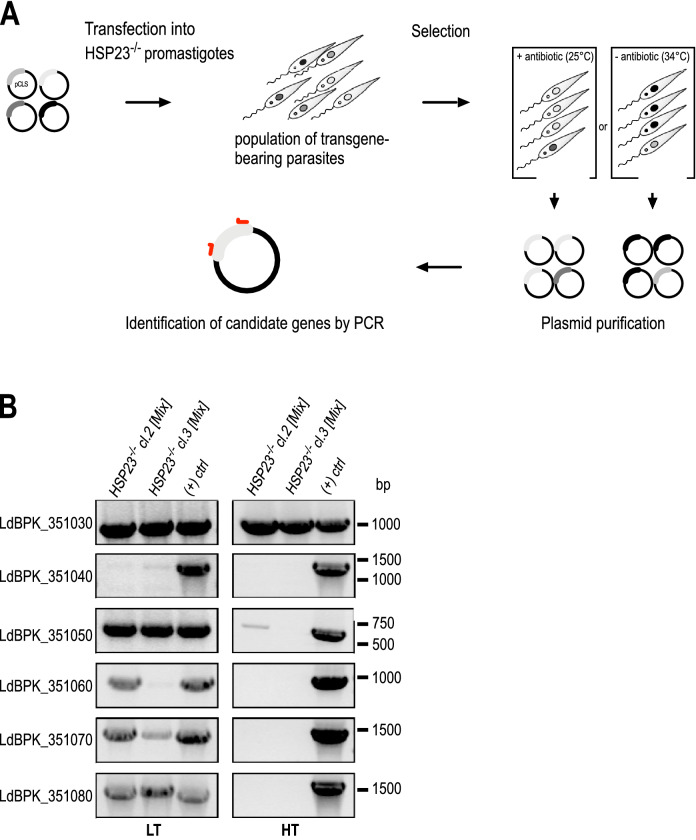
Selection of candidate genes in the HSP23^−/−^ background at non-permissive temperatures (**A**) Schematic drawing of the transgene selection procedure. Six over expression plasmids harbouring LdBPK_351030 to LdBPK_351080 were co-transfected into HSP23^−/−^ null mutants and selected either at LT with the selective antibiotic, or at HT, generating HSP23^−/−^ [Mix]-LT or HSP23^−/−^ [Mix]-HT cell populations. After selection, plasmids were isolated from the *Leishmania* populations by Phenol–Chloroform extraction. (**B**) Analytical PCR using gene-specific primers to test for the presence of candidate transgenes in the recovered plasmids. Transgene plasmid DNA was used as positive control (( +) ctrl). PCR products were analysed by agarose gel electrophoresis and ethidium bromide staining; the position of corresponding DNA size marker bands is shown on the right. The left panel shows the PCR products after LT selection, the right panel shows the results after HT selection.

Next, we tested whether the escape mutant phenotype can be attributed to the observed amplification of the six-gene cluster on chromosome 35. We therefore co-transfected a mixture of six different over expression plasmids, harbouring the genes from the amplified cluster (LdBPK_351030—LdBPK_351080), into TS HSP23^−/−^ lines. The transfected lines were selected either at LT under antibiotic selection or at HT without antibiotics, herein named as HSP23^−/−^ [Mix]-LT and HSP23^−/−^ [Mix]-HT, respectively (Fig. 5A). While empty vector-transfected cells did not survive the selection at non-permissive temperatures, two independent TS HSP23^−/−^ clones transfected with the plasmid mixture recovered, confirming that over expression of genes from the amplified cluster causes the escape mutant phenotype.

To pinpoint the gene(s) mediating survival at HT, plasmid DNA was re-isolated from surviving populations, and PCR was performed with transgene-specific primers (Fig. 5B). While a complete set of plasmids was still recovered from HSP23^−/−^ [Mix]-LT after LT selection (LTS), selection at HT (HTS) almost exclusively selected for the CK1.2 transgene, confirming a role of CK1.2 in reversing the TS phenotype of HSP23^−/−^. This was confirmed by qRT-PCR analysis, showing only increased abundance of CK1.2 RNA after HT selection, but not of the other transgenes (Fig. S4).

### CK1.2 transgene is amplified under temperature selection

**Figure 6:**
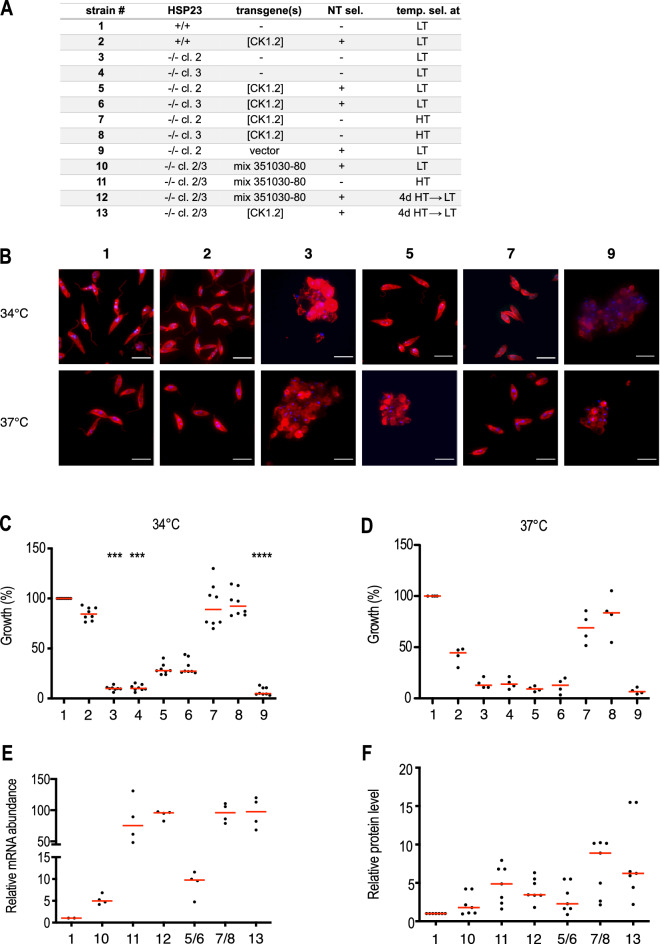
Characterisation of transgenic *L. donovani* strains. (**A**) List of transgenic parasite cell lines tested. NT sel = selected under nourseothricine; temp. sel. = temperature selection scheme. (**B**) 5✕10^5^ cells/ml were seeded into 7 ml of M199 + medium and incubated for 4 days at 34 °C or 37 °C. On day 4, cells were stained with mouse anti-α-tubulin mAB (1:4000) and anti-mouse-IgG Alexa Fluor 594 (1: 500) and DAPI. Images were processed and merged using the ImageJ Fiji Software (Version 2.0.0). Scale bar:10 µm (**C**, **D**) Growth behaviour of transgenic *L. donovani* strains at 34 °C (n = 8) or 37 °C (n = 4). Cells were seeded at 5✕10^5^/ml and grown for 4 days. Cell density was measured and normalised to wild type (HSP23^+/+^) cell density. Significance was tested using the Kruskal–Wallis test: ***p < 0.001, ****p < 0.0001. (**E**, **F**) CK1.2 expression levels were determined by qRT-PCR using gene-specific primers (**E**) and Western blotting using the anti-CK1.2 antibody (**F**). Data were normalised to the HSP23^+/+^ samples.

To further validate theses findings, we tested whether transfection of the CK1.2-encoding over expression plasmid alone can restore temperature tolerance to HSP23^−/−^. Indeed, ectopic over expression of CK1.2 alone abrogated characteristic heat-induced defects such as the aggregation and rounding of the normally elongated promastigotes, as visualised by anti-alpha-tubulin staining (Fig. 6B), but also restored tolerance to EtOH and H_2_O_2_-induced protein folding stress at LT (Fig. S5).

Moreover, in all tested strains (Fig. 6A), ectopic expression of CK1.2 strongly depended on the pre-selection condition: HT-selected lines were able to grow both at 34 °C and 37 °C and reached similar cell densities as HSP23^−/−^ esc mutants (Fig. 6C,D). In contrast, only partial reversion of the TS phenotype was observed in LT-selected HSP23^−/−^ [CK1.2] lines as judged by the comparably slow growth at 34 °C and failure to grow at 37 °C (Fig. 6C,D).

Based on these results, we reasoned that the compensation for the loss of HSP23 might correlate with CK1.2 expression levels. To test this hypothesis, we analysed CK1.2-encoding mRNA as well as protein levels in the over expressing lines selected under both permissive and non-permissive conditions. As suspected, HTS resulted in ~10–15-fold higher RNA and ~2.5-fold higher CK1.2 protein levels when compared to LTS (Fig. 6E,F). Further, shifting HSP23^−/−^ [Mix]-LT and HSP23^−/−^ [CK1.2]-LT lines to higher temperatures was associated with a 10–19-fold increase of CK1.2 mRNA levels and 2.6–3.9-fold protein levels. Continuous incubation of HSP23^+/+^ wild type cells at non-permissive temperatures or transfection of HSP23^+/+^ parasites with CK1.2 over expression plasmids with subsequent HTS did not result in comparable increases of CK1.2 mRNA or protein levels (data not shown). In addition, the increased mRNA as well as protein abundance observed for the heat-selected HSP23^−/−^ esc1/2 populations was reversible once the cultures were transferred back to LT (Fig. S6).

In summary, our data demonstrate that i) there is a strong selective pressure for increased CK1.2 protein levels in HSP23^−/−^ null mutants and ii) that the observed gene dosage changes are under tight, temperature-dependent selection.

### HSP23 is a Casein Kinase 1.2 substrate in vitro

**Figure 7:**
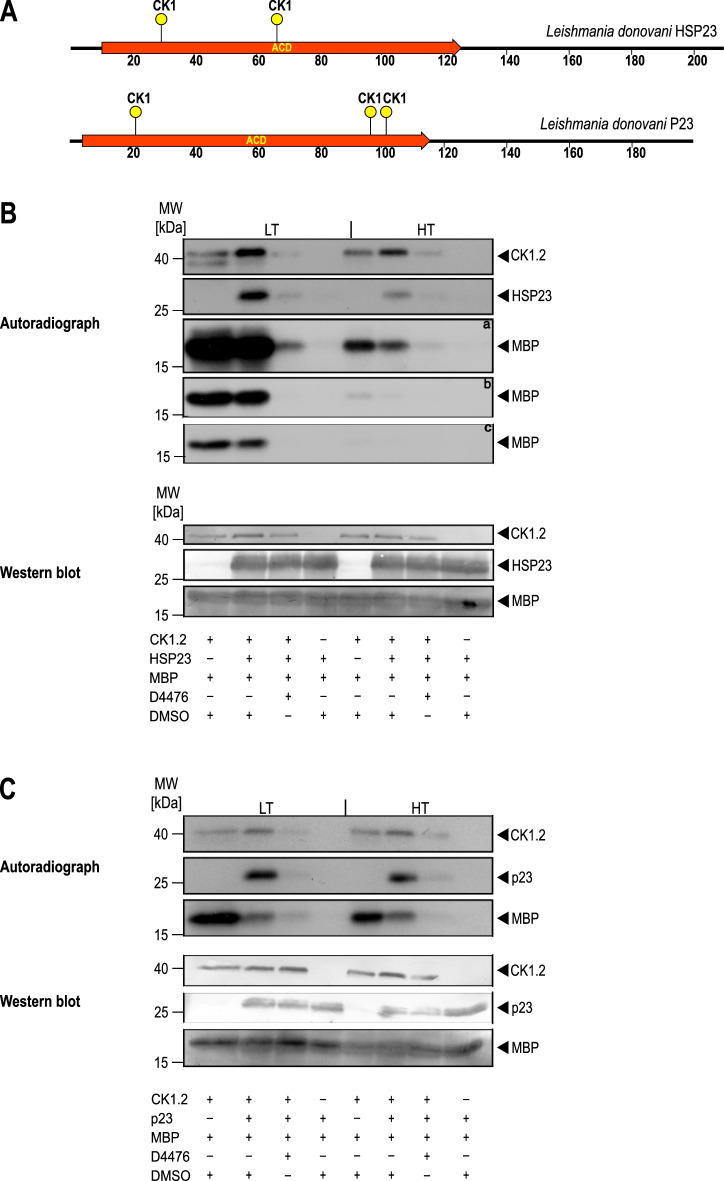
In vitro kinase assays using [*γ*-^32^P]-ATP. (**A**) Schematic representation of the putative CK1 phosphorylation sites present in HSP23 and P23. (**B**, **C**) 0.25 µg recombinant Casein kinase 1.2 was incubated with 3 µg of myelin basic protein (MBP), 1.57 µg of recombinant HSP23 (**B**) or 1.3 µg P23 (**C**) in the presence of either 10 µM D4476 inhibitor or equivalent concentrations of DMSO. The reactions were incubated at LT (25 °C) or HT (37 °C) for 30 min and stopped by addition of loading buffer. A reaction without the kinase was included to test for possible autophosphorylation of substrates. Proteins were separated on a 12.5% SDS-PAGE gel followed by Western blot. The incorporation of [*γ*-^32^P]-ATP was monitored by autoradiography (upper panels). a = 1 week exposure; b = 24 h exposure, c = 12 h exposure of X-Ray films. The blot was probed with an anti-CK1.2 (SY3535), HSP23 and P23 antibody, or stained with Coomassie blue to evaluate loading (lower panels). The figure shows representative Western blots from a series (n = 4) of experiments.

Casein kinase 1.2 is implicated in the heat shock response of *Leishmania* as an upstream protein kinase of HSP90^45^. Moreover, both HSP23 and the closely related, HSP90-modulating co-chaperone P23^43^ contain possible CK1 phosphorylation sites (Figs. 7A, S7). To elucidate possible enzyme–substrate interactions between CK1.2 and HSP23, but also P23, we performed in vitro phosphorylation assays with recombinantly expressed *L. donovani* CK1.2 using myelin basic protein (MBP), HSP23 and P23 as substrates and/or modulatory chaperones. The proteins were incubated for 30 min with [*γ*-^32^P]-ATP at LT and HT, separated by SDS-PAGE and visualised by autoradiography.

Figure 7B shows the activity of CK1.2 in a reaction mix containing the kinase, MPB and HSP23. CK1.2 readily phosphorylates MBP at LT and at HT, with a slight drop of activity at HT. Adding HSP23 to the mix has a threefold effect: i) autophosphorylation of CK1.2 increases, ii) HSP23 becomes radiolabeled, and iii) MPB phosphorylation is reduced at higher temperature. We conclude that CK1.2 autophosphorylation and the concomitant reduction of activity is furthered by HSP23. In turn, HSP23 is a substrate of CK1.2. The latter activity is verified by the loss of HSP23 phosphorylation in the presence of the CK1-specific inhibitor, D4476.

We also tested the impact of the co-chaperone P23 on CK1.2 activity. As shown in Fig. 7C, P23 has a very similar impact to HSP23, furthering autophosphorylation of CK1.2 and reducing MBP phosphorylation. Like HSP23, P23 is a substrate for CK1.2-mediated phosphorylation, adding a third member of the chaperone family of proteins to the list of CK1.2 clients.

We conclude, that CK1.2 over expression has a likely effect on the phosphorylation status and possibly the activity of chaperones in *Leishmania* and its over expression by gene amplification may thus impact on the protein folding homeostasis in the parasite.

## Discussion

### Genetic compensation for the loss of HSP23 in *Leishmania*

Double-allele replacement of *HSP23* in *L. donovani* results in a reduced growth at 25 °C, the temperature commonly associated with the promastigote stage, but also a loss of viability at temperatures > 30 °C^42^. Our results show that all HSP23^−/−^ null mutants that we obtained carry a partial or complete amplification of chromosome 35, either as trisomy or as a duplication of the 5′-terminal third of this chromosome. A passage at 37 °C of the HSP23^−/−^ esc0 clone, which overcame the growth reduction at 25 °C, even selected for the massive amplification of a six-gene region from the 5′ terminus of chromosome 35. As reported before^16,23^, changes in DNA copy numbers observed for the six-gene cluster on chromosome 35 correlated well with an increase of mRNA levels. However, upon removal of HTS, mRNA returned to basal levels indicating that the gDNA amplificates are lost from the selected populations in the absence of temperature selection, although this remains to be verified experimentally.

Our observations match the genomic changes associated with drug resistance where selective chromosomal somy changes appear early in the development of resistance and are followed, upon more stringent selection, by the appearance of extrachromosomal amplificates that are usually lost from the populations once the selection is removed^18–20,53–55^.

In recent years, remarkable progress has been made towards understanding the mechanisms underlying the high plasticity of the *Leishmania* genome. Circular or linear extrachromosomal episomes are commonly formed through rearrangements at the level of direct or inverted repeat sequences^56,57^. Furthermore, short interspersed degenerated retroposons (SIDERs) that are widely distributed through the entire *Leishmania* genome were shown to be involved in the regulation of gene expression at both the post-transcriptional (SIDER2) or translational (SIDER1) level^58,59^ but also in inducing DNA rearrangements^19^.

Indeed, a genome-wide bioinformatic analysis of the *Leishmania* genome^19^ described several SIDER sequences that either flanked or were present within the identified DNA region on chromosome 35 amplified in heat selected HSP23^−/−^ esc mutants. More importantly, the amplification identified by NGS in the HSP23^−/−^ esc1,2 mutants matched with a predicted putative product, thus highlighting the power of SIDER element-based prediction of DNA rearrangements and its importance in the response to changing stress conditions.

### Over expression of Casein Kinase 1.2 rescues temperature tolerance in HSP23^−/−^

Two lines of evidence point at amplification of casein kinase 1.2 as the mechanism for restoring temperature tolerance to HSP23^−/−^ mutants. Firstly, of the six genes found on the amplified gene cluster after HTS and transfected as transgenes into HSP23^−/−^ clones 2 and 3, only LdBPK_351030 – CK1.2 was retained under HTS of the mixed population while LTS yielded all six transgenes. Secondly, in the CK1.2-over expressing HSP23^−/−^ parasites, survival and growth under HTS correlated with CK1.2 mRNA and protein abundance, resulting in almost wild type-like temperature tolerance for HT-selected populations with 6–ninefold increased CK1.2 abundance. Shifting the LT-selected strains to HT quickly led to an increase of expression. In a similar way, HT-selected HSP23^−/−^ esc strains that were transferred back to LT, showed a reduction of CK1.2 mRNA and protein suggesting that the observed gene amplification is reversible. We conclude that survival and growth of HSP23^−/−^ mutants at temperatures matching mammalian tissue temperature is a function of CK1.2 expression. Conversely, cultivation at ambient temperature leads to a rapid reduction of over expression, indicating adverse effects of increased CK1.2 abundance in the absence of temperature stress.

Members of the casein kinase 1 family are highly conserved acidophilic serine/threonine kinases with reported roles in cell cycle^60^, circadian rhythm^61–63^, cell signalling, and membrane trafficking^64^. The *Leishmania* genome harbours six different Casein kinase isoforms with CK1.2 being the most highly expressed and abundant form^65–67^. CK1.2 was shown to display an ubiquitous localisation and was found in the cytoplasm, nucleus, the basal body and the flagellar pocket of *L. donovani*^68^, the major interface of the parasite with its environment and the origin of exosomes. Consistently, CK1.2 is known to be part of the protein load of immune-modulatory exosomes that are shed by *Leishmania* parasites in a temperature-dependent manner during the intracellular amastigote stage within the host cell^69,70^. This, and the fact that inhibition of CK1.2 with the specific inhibitor D4476 is cytotoxic in axenic amastigotes and reduces the parasite load of infected macrophages in vitro^52^, highlights the essential role of this protein kinase in stage differentiation and intracellular survival.

As reported previously for human CK1 enzymes^71^, known substrates of *Leishmania* CK1.2 include the major chaperones HSP70 and HSP90, the latter being essential for the rapidly growing promastigote and playing a pivotal role in the life cycle control of *Leishmania donovani*^45,68,72,73^.

### CK1.2 phosphorylates HSP23 and P23 in vitro

Our results indicate that another two chaperone proteins, HSP23 and P23, are likely substrates of CK1.2. Indeed, both proteins contain several canonical consensus sequences (pSer/Thr-X-X-(X)-Ser/Thr) for CK1 protein kinases (Fig. 7A). P23 was already described to be phosphorylated by CK2 in other organisms^74,75^. Moreover, *Leishmania* HSP23 and P23 appear to modulate the CK1.2 activity and specificity in turn (Fig. 7B,C), increasing autophosphorylation activity and reducing MBP phosphorylation. The inverse correlation between autophosphorylation and substrate-specific activity is a known characteristic of CK1 in other systems^76–78^.

P23 is a known co-chaperone of HSP90 and binds to the middle domain of HSP90 with a higher affinity for the ATP-bound state. This interaction was shown to temporarily stabilise the closed conformation of HSP90 and at the same time slowing the ATPase cycle^79–84^. Such an inhibitory effect on the HSP90 ATPase activity was also described for P23 and, to a lesser degree, for HSP23 in *Leishmania braziliensis*^85^. Strong structural as well as functional conservation of HSP23 orthologs in Trypanosomatida (V. Adaui, C. Kröber-Boncardo, unpublished) suggests a similar modulating effect of the *L. donovani* HSP23 protein on HSP90 function.

The interaction between P23 and HSP90 can be inhibited by the HSP90 specific inhibitors geldanamycin (GA) and radicicol leading to the abrogation of a functional HSP90 cycle^86,87^. The loss of P23 in *Leishmania,* but also in other organisms, consistently results in hypersensitivity to those HSP90 inhibitors. The fact that over expression of HSP23 in *L. donovani* can compensate the loss of P23 under GA or radicicol induced HSP90 inhibition^43^ further supports data demonstrating an intrinsic co-chaperone activity of HSP23 on HSP90. The growth reduction phenotype observed for HSP23^−/−^ null mutants may therefore be partly due to a deregulation of HSP90. Since HSP90 is also a known in vitro substrate of CK1.2^45^, the over expression of CK1.2 in HSP23^−/−^ escape variants might be one way to counteract the deregulation by altering the phosphorylation state of HSP90 itself or of its co-chaperones such as P23. Post-translational modifications of HSP90 are extremely important for the fine-tuning of the chaperone cycle and affect its conformation, ATPase activity, interaction with co-chaperones, the recruitment and activation of various client proteins, cell growth, and viability^45,71,88^.

A surprising result was obtained with a gene add-back line of HSP23^−/−^ which showed restored in vitro growth at 37 °C and under 2% ethanol challenge, but was unable to proliferate under H_2_O_2_, while the complementation with CK1.2 alleviated H_2_O_2_-mediated growth inhibition. We suspect that the effects of HSP23^−/−^ on growth under H_2_O_2_ mirror those at 25 °C standard culture conditions where gene add-back also had a moderate effect. This indicates that the strong growth defects under heat stress inducing conditions, i.e. HT or 2% ethanol, and the more moderate effects seen at LT and under oxidative stress may be due to different cellular pathways.

In summary, our data suggest a regulatory network comprising CK1.2, HSP23, P23 and HSP90. This is supported by the following results : i) HSP23, P23, and HSP90 are in vitro substrates of CK1.2 and ii) HSP23 and P23 affect CK1.2 activity but also HSP90 activity^85^.

Comparative phosphoproteome analysis of HSP23^−/−^ esc mutants and CK1.2 over expressing lines under both permissive and non permissive temperatures may provide new insights into the functional network of these chaperones and their regulation by CK1.2. In addition, such studies may allow to identify novel clients and downstream signalling pathways of CK1.2 which may elucidate the effect of CK1.2 over expression in HSP23^−/−^ null mutants and eventually shed light on its role in *Leishmania* temperature tolerance. Furthermore, the putative CK1.2 phosphorylation site(s) of HSP23 but also P23 can be mutated to assess the resulting phenotype in terms of in vitro growth, temperature- and chemotolerance.

### Impact on target-specific drug development

Our data show that in spite of its importance for the parasite’s temperature tolerance, HSP23 may be a poor target for therapeutic intervention due to the prevalence of genetic variants that can overcome the loss of HSP23 function. The plasticity of the *Leishmania* genomes offers large ranges of genetic variants from which to select, both in the laboratory and in the wild, allowing them to overcome the loss or loss of function of single proteins. Thus, our data also highlight the risk of using genetically modified, live-attenuated *Leishmania* parasites^89–91^ for vaccine purposes, since supposedly attenuating genetic modifications may be easily reversed or compensated through selection from pre-existing, beneficial gene amplification variants within *Leishmania* populations.

## Materials and methods

### Reagents and chemicals

All reagents, unless otherwise stated, were purchased from Merck, Darmstadt, Germany or its Sigma-Aldrich subsidiary.

### *Leishmania* culture conditions

*Leishmania donovani* strain 1S^36^ and mutants thereof were cultured in M199 + medium^73^ with the respective antibiotics at 25 °C and passaged every 3–4 days.

### Electrotransfection of *Leishmania* parasites

Electrotransfection and selection was performed as described^92^. To isolate single clones from a transgenic parasite population, 0.5 cells per well were seeded in 96-well plates in a final volume of 200 µl M199 + medium supplemented with the respective antibiotics G418 (50 µg/ml, Roth, Karlsruhe, Germany), puromycin (25 µg/ml) and ClonNat (150 µg/ml, Werner Bioreagents, Jena, Germany) and 1 × Penicillin/Streptomycin. After 10–14 days of selection, emerging clones were transferred to culture flasks and further passaged under antibiotic selection.

### Construction and preparation of recombinant DNA

LdBPK_340230 (*HSP23*), LdBPK_351030 (*CK1.2*), LdBPK_351040, LdBPK_351050, LdBPK_351060, LdBPK_351070 and LdBPK_351080 coding sequences were amplified from *L. donovani* 1S genomic DNA using specific primer pairs (Table S1) that introduce restriction sites as indicated. Fragments were subsequently ligated into the pCL2S vector^93^ predigested at the matching restriction sites. Plasmids were amplified in *E. coli* DH5α and purified by CsCl density gradient ultracentrifugation^94^.

For generation of the CK1.2 protein expression plasmid, the pJC45 plasmid^40^ was modified by introducing a *Kpn*I restriction site into the multiple cloning site to create the pJC65 plasmid using primer Link-pJC65 + and Link-pJC65-. The open reading frame of *Leishmania donovani* CK1.2 was isolated from the pCL2S_LdBPK_351030 plasmid using *Kpn*I and *Bam*HI restriction sites and ligated between the matching sites in pJC65. A map of pJC65 is shown in Fig. S8.

### Plasmid purification from *Leishmania* parasites

2–4 ✕ 10^9^ log-phase promastigotes previously transfected with a mixture of over expression plasmids were harvested by centrifugation at 1250 ✕ g for 8 min at 4 °C. After three washing steps with 1✕ PBS, plasmids DNA was purified by alkaline lysis following the protocol for plasmid DNA mini preparation^94^. After phenol/chloroform/isoamylalcohol (25:24:1) extraction, plasmid DNA was precipitated by addition of 0.1 volume of 10 M ammonium acetate and 2.5 volumes 100% Ethanol. The pellet was dissolved for 24 h at 4 °C with Tris/EDTA buffer (pH 8.0). Next, 10 µl (approximately 100 ng) of isolated plasmid DNA was electroporated into *E.coli* XL one Blue Electroporation-Competent Cells (Agilent Technologies, Santa Clara, USA) cells using 0.1 cm gap Gene Pulser cuvettes (Bio Rad, Munich, Germany). Amplified DNA was isolated using the NucleoBond PC 20 kit (Machery-Nagel, Düren, Germany) following the manufacturer’s instructions, and eluted in 20 µl ultrapure ddH_2_O.

### Next generation sequencing

DNA libraries were created using the Nextera XT library kit (Illumina, San Diego, USA) and the Nextera XT index kit (Illumina, San Diego, USA) according to manufacturer’s instructions. *Leishmania* gDNA was isolated using the Bioline Isolate 2 Genomic DNA Kit according to the manufacturer's protocol and set to a final concentration of 0.5 ng/µl using the Qubit (3.0)-system (Thermo Fisher Scientific, Waltham, USA). 1 ng of gDNA was used in the subsequent transposase-mediated tagmentation step. After purification of the DNA library using the Agencourt AMPure XP kit (Beckman Coulter, Fullerton, USA), the size distribution of DNA fragments was determined using a Bioanalyzer (Agilent Technologies, Santa Clara, USA) and the high sensitivity DNA analysis kit (Agilent Technologies, Santa Clara, USA). Each sample was further diluted to 4 nM taking into account the average fragment size and DNA concentration. A DNA library pool was prepared mixing 5 µl of each diluted sample in a Lobind 1.5 mL tube (Eppendorf, Hamburg, Germany). To denature the DNA-library, 5 µl of the DNA pool was mixed with 5 µl of freshly prepared 0.2 N NaOH. After incubation for 5 min at RT, 990 µl of pre-chilled HT1 buffer was added resulting in a 20 pM DNA library. The DNA library was further diluted with HT1 buffer to 10 pM in a final volume of 600 µl and 6 µl of a denatured PhyX reference library was added. Prior to loading the sequencing cartridge MiSeq Reagent Kit v3 (600-cycle) the library was heat denatured (95 °C, 2 min) and incubated for 5 min on ice. Sequencing was carried out on a MiSeq sequencer (Illumina, San Diego, USA).

### RNA extraction, cDNA synthesis and quantitative Real-time PCR (qRT-PCR)

Total cellular RNA was isolated from 5 ✕ 10^7^ log-phase *L. donovani* parasites using the InviTrap Spin Cell RNA Mini Kit (Stratec, Birekenfeld, Germany) according to manufacturer‘s instructions. First strand cDNA synthesis from 800 ng of RNA was performed using the QuantiTect(R) Reverse Transcription Kit (Qiagen, Venlo, Netherlands) following the manufacturer's protocol.

For qRT-PCR, 1 µl of cDNA was included in a final volume of 20 µl using the Biozym Blue S'Green qPCR Kit (Biozym Scientific GmbH, Oldendorf, Germany). Reactions were run on a Rotor Gene real time PCR cycler (RG 6000, Corbett Research, Sydney, Australia) and analysed^95^. Gene expression levels were normalised to HSP23^+/+^-LT samples. qPCR primer sequences are listed in Table S1.

### Western blotting

For each sample, 1 ✕ 10^7^ parasites were dissolved in sample buffer and separated by SDS-PAGE, followed by semi-dry Western blot^94^. Primary antibody polyclonal anti-CK1.2 rabbit (SY3535) (1:250)^52^ or polyclonal anti-HSP70 IgY(1:500)^38^, anti-P23 IgY (1:500)^43^ and anti-HSP23 IgY (1:250)^42^ in blocking solution was used in conjunction with anti-rabbit-IgG::-AP (goat, 1:1000) or anti-chicken-IgY::AP (rabbit, 1:5000) secondary antibodies (Dianova, Hamburg, Germany) and developed using Nitro Blue tetrazolium chloride and 5-bromo-4-chloro-3-indolyl phosphate^39^.

### Immunofluorescence assays

1 ✕ 10^7^ parasites were gently sedimented (10 min, 1000 ✕ g, 4 °C), washed once with PBS and suspended in 100 µl of PBS. A 40 µl aliquot was smeared on a glass microscopic slide, air-dried and fixed for 3 min in ice cold methanol. Slides were gently washed three times (0.1% Triton X-100 in PBS) and permeabilised for 15 min in 0.1% Triton X-100, 50 mM NH_4_Cl in PBS. Slides were then incubated in blocking solution (2% BSA, 0.1% Triton X-100 in PBS) for 1 h. Polyclonal anti-tubulin-IgG (mouse) (1:4000) was added in blocking solution and incubated for 1 h at RT. Cells were washed three times for at least 15 min and then incubated with anti-mouse Alexa Fluor(R) 594 IgG (goat, 1:250)) together with DAPI (1:50 in PBS). For microscopic visualisation, slides were covered with 20 µl of Mowiol and cover slips and left for 24 h at 4 °C. Fluorescence microscopy was carried out using an EVOS FL Auto Cell Imaging System (Thermo Fisher Scientific, Waltham, USA). Images were processed using the ImageJ, Fuji Software (Version 2.0.0).

### Expression and purification of recombinant HSP23 and CK1.2 protein

The pJC65-CK1.2, pJC45-HSP23^42^ and pJC45-P23^43^ plasmids were transformed into the bacterial strain BL21(DE3) (pAPlacIQ)^40^ and soluble (His)_10_-tagged proteins were purified using HisBind Resin (Novagen, Madison, WI) as described previously^96^. The eluate was supplemented with 10% glycerol, shock-frozen and stored in aliquots at − 80 °C.

### Casein kinase assay

CK1.2 Kinase in vitro activity testing was performed essentially as described^45^. Briefly, recombinant proteins were added to buffer A (40 mM HEPES (pH7.5), 130 mM KCl, 15 mM MgCl_2_, 0.5 mM 1,10-Phenanthroline, 1 mM Phenylmethylsulfonyl fluoride, 25 mM ß-glycerophosphate, 0.1 mM sodium orthovanadate, 1 mM EGTA, 1 mM dithiothreitol, 0.05 mM ATP and 10 µCi [*γ*-^32^P]-ATP (Hartmann Analytics, Braunschweig, Germany) in a final volume of 25 µl. Recombinant proteins and substrates were used in the following concentrations: recLdCK1.2: 0.25 µg (6 pmol), recLdHSP23: 1.57 µg (60 pmol), recLdP23: 1.3 µg (60 pmol), myelin basic protein (MBP): 3 µg (162 pmol). CK1.2 specific inhibitor D4476 was used at a concentration of 10 µM in the presence of 0.7% DMSO. Controls were performed with equivalent final concentrations of DMSO. The reactions were incubated for 30 min at 25 °C or 37 °C and stopped by adding 1 Vol of SDS-PAGE sample buffer^94^. The samples were heated to 95 °C for 10 min and run on a 12.5% SDS polyacrylamide gel. After Western blot onto a polyvinylidenefluoride (PVDF) membrane (GE Healthcare Life Sciences, Munich, Germany), ^32^P incorporation was detected by autoradiography (Fuji Super RX-N, Tokyo, Japan).

### In silico procedures

In silico cloning, DNA and protein sequence analysis was performed using the MacVector software version 17.x. Numerical data were analysed using the Prism software (version 8, GraphPad Software LLC). Halftone images were optimised *in toto* for contrast and brightness using the ImageJ Fiji Software (Version 2.0.0)(NIH). Composite figures were assembled using the Intaglio software (Purgatory). The heat map was generated using the Heatmapper Online Tool^97^.

### Bioinformatics and NGS analysis

Reads were aligned to TriTrypDB version 42 of *L. donovani* BPK using Bowtie2^98^. Chromosome ploidy was determined by an iterative approach using an in-house program: initially, the coverage of each chromosome was estimated to be 2.0. The mean coverage of the positions in the 15th to 85th quantile and the ratio coverage/mean were calculated. Coverages were then updated according to these fractions and the procedure repeated until conversion. For gene coverages, a similar approach was used, with the difference that the copy number was normalised to the previously calculated chromosome ploidies.

### Statistics

Statistical comparisons between groups were performed using one-way analysis of variance (ANOVA)/Kruskal–Wallis test with Dunn’s post test. Differences were considered significant at a level of *p* < 0.05.

## Supplementary information

Supplementary Information 1.

Supplementary Information 2.

## Data Availability

Whole genome sequencing data are available from: https://www.ncbi.nlm.nih.gov/sra/PRJNA633969.
