# Supplementary material for: Casein kinase 1.2 over expression restores stress resistance to Leishmania donovani HSP23 null mutants
Source: Sci Rep. 2020 Sep 29;10:15969. doi: 10.1038/s41598-020-72724-x (PMC7525241; doi:10.1038/s41598-020-72724-x)
Supplement: Supplementary file 1 — Supplementary Information 1. [file 41598_2020_72724_MOESM1_ESM.pdf]

Supplementary Information

Casein Kinase 1.2 Over Expression Restores Stress Resistance to *Leishmania donovani* HSP23 Null Mutants

Constanze Kröber-Boncardo[1], Stephan Lorenzen[2], Christine Brinker[1], and Joachim Clos[1*]

[1]Leishmania Group and [2]Dept. of Epidemiology, Bernhard Nocht Institute for Tropical Medicine, Hamburg, Germany

* Correspondending author:

Joachim Clos, Bernhard Nocht Institute for Tropical Medicine, Bernhard Nocht St 74,

D-20359 Hamburg, Germany, email clos@bnitm.de, phone +49 40 42818 481

Supplementary Information

Table S1

Primers used for CK1.2 study	Primer sequence 5'-3'
**qPCR**	
LdBPK_340230.1_fwd	CCATTCCTGGATTTGGCTCGG
LdBPK_340230.1_rev	GCATATCGCCGTCGTCATCTCC
LdBPK_351030.1_fwd	CACGTGTACATCATCGACTTTGG
LdBPK_351030.1_rev	CTCTTGCCTTCCTTGTATGGGAT
LdBPK_351040.1_fwd	ACACTCTCCCTTCGCATTATCAG
LdBPK_351040.1_rev	CTCGATGAAGTAGATCGGTTGCT
LdBPK_351050.1_fwd	TTTTGGATTAAGCGCCTGCAAC
LdBPK_351050.1_rev	GAAAGTAAATGGTCGTGTAGGCG
LdBPK_351060.1_fwd	CAGTACTACACACACAACCCGTA
LdBPK_351060.1_rev	CGACAACTTCTTGGTAGACTGGA
LdBPK_351070.1_fwd	TAGTCTTCTACGTGCTTCTCTGC
LdBPK_351070.1_rev	GTGATTTGGGTTGAAGAGGACAC
LdBPK_351080.1_fwd	CGAGTACGGCAACTTCAACGATA
LdBPK_351080.1_rev	CCGGTCGTCTATGTCATCTTCTT
**over expression plasmids construction**	
351030_KpnI_fwd	GGAGGGTACCATGAACGTGGAACTGCGCGTC
351030_NdeI_rev	GGAGCATATGCTACTGCTGTTCTTGCGCAC
351040_KpnI_fwd	GGAGGGTACCATGCACACGCCACCCCTCTC
351040_NdeI_rev	GGAGCATATGTCACATGAAGCGAGCCGCCA
351050_KpnI_fwd	GGAGGGTACCATGACCTCCCCGGCAGCCGC
351050_NdeI_rev	GGAGCATATGCTAGATTCCTGTTGTCTGTAAG
351060_NdeI_fwd	GGAGCATATGATGACGAGCCAGTCGCACGTG
351060_BglII_rev	GGAGAGATCTTTAGGGCTCCATCACCATCG
351070_KpnI_fwd	GGAGGGTACCATGACCGTTCTGCCACCCAAG
351070_NdeI_rev	GGAGCATATGTCACTTGCGCAAAGCGTCGG
351080_KpnI_fwd	GGAGGGTACCATGGGTAAGCGAAACGAAAG
351080_NdeI_rev	GGAGCATATGCTACATGGATGCGACCACGC
**pJC65 multiple cloning site**	
Link-pJC65+	CATGGGCCATCATCATCATCATCATCATCATCATCACGGTGGTACCA
Link-pJC65-	AGCTTGGTACCACCGTGATGATGATGATGATGATGATGATGATGGCC

Supplementary Information

Table S2: Ploidy values for chromosomes based on NGS sequence read coverages.

CHR	HSP23+/+ #1-LT	HSP23+/+ #2-LT	HSP23+/+ #1-HT	HSP23+/+ #2-HT	HSP23-/- cl.2-LT	HSP23-/- cl.3-LT	HSP23-/- esc #1-LT	HSP23-/- esc #2-LT	HSP23-/- esc #1-HT	HSP23-/- esc #2-HT
1	1.91	1.61	1.56	1.63	1.85	1.89	1.66	1.70	1.74	1.69
2	2.12	1.95	1.96	2.06	2.35	2.20	2.03	2.09	2.13	2.00
3	1.80	1.78	1.87	1.81	1.97	2.02	1.69	1.84	1.86	2.37
4	1.70	1.79	1.80	1.80	1.98	1.93	1.87	1.85	1.87	1.83
5	1.82	1.74	1.73	2.22	1.86	2.02	1.77	1.87	1.85	1.78
6	1.95	1.95	1.94	1.96	2.06	2.10	1.93	1.98	2.06	1.95
7	2.07	1.92	1.95	1.93	2.08	2.00	1.98	1.98	2.05	1.96
8	2.66	2.78	2.00	2.48	3.06	3.07	2.92	2.84	2.79	2.55
9	1.77	1.86	1.80	1.76	1.97	1.94	1.88	1.92	1.88	1.87
10	1.94	1.99	2.01	1.97	2.07	2.08	1.96	1.99	1.97	1.99
11	2.09	2.14	2.26	2.12	2.05	2.09	2.15	2.05	2.08	2.12
12	3.04	3.11	2.92	2.51	3.03	3.01	2.05	1.99	2.01	2.05
13	2.02	1.90	2.10	1.90	1.97	1.91	1.96	1.96	1.92	1.88
14	1.82	1.86	1.95	2.22	1.97	1.91	2.71	2.71	2.66	2.02
15	1.94	1.84	1.97	2.17	1.96	1.91	1.98	1.97	1.92	1.86
16	1.99	2.01	2.04	1.97	2.19	2.03	2.07	2.10	2.07	2.08
17	2.10	2.05	1.99	1.96	2.09	2.02	1.98	2.05	2.05	1.99
18	2.02	2.07	2.11	2.29	1.98	1.93	1.99	2.00	1.96	1.98
19	1.93	1.98	1.96	1.96	1.97	2.00	2.06	1.97	1.96	1.93
20	1.95	2.06	1.84	1.50	2.08	2.02	2.00	1.96	2.01	2.04
21	1.98	1.99	2.04	2.01	2.07	2.01	1.99	2.02	2.02	2.00
22	2.01	1.98	1.99	2.01	2.06	2.01	2.00	2.00	1.99	1.95
23	3.11	3.22	3.25	2.67	3.15	3.16	2.18	2.08	2.11	2.14
24	2.01	1.96	2.00	2.01	1.98	1.93	1.98	2.00	1.98	1.98
25	1.92	1.99	1.98	2.00	1.99	1.94	1.99	1.97	2.01	1.97
26	2.95	3.05	2.98	2.82	3.15	2.87	3.11	3.03	3.02	3.01
27	2.14	2.16	2.17	2.00	2.07	2.01	2.17	2.06	2.05	2.07
28	1.96	2.11	2.17	2.00	2.00	1.94	2.10	2.05	2.04	2.03
29	2.17	2.18	2.12	2.00	1.99	2.01	2.09	2.05	2.01	2.04
30	2.10	2.20	2.19	2.01	1.98	2.00	2.17	2.05	2.05	2.12
31	4.11	4.30	4.37	4.07	4.16	3.88	4.32	4.16	4.17	4.18
32	2.12	2.17	2.18	2.02	2.00	1.94	2.18	2.02	2.09	2.08
33	2.09	2.16	2.17	2.03	2.06	1.94	2.17	2.00	1.97	2.01
34	2.09	2.12	2.11	1.96	1.98	1.92	2.16	2.00	1.94	1.97
35	2.11	2.13	2.13	1.91	2.93	2.87	2.36	2.26	1.98	2.02
36	1.95	2.16	2.19	1.91	1.97	1.91	2.16	2.00	1.98	2.05

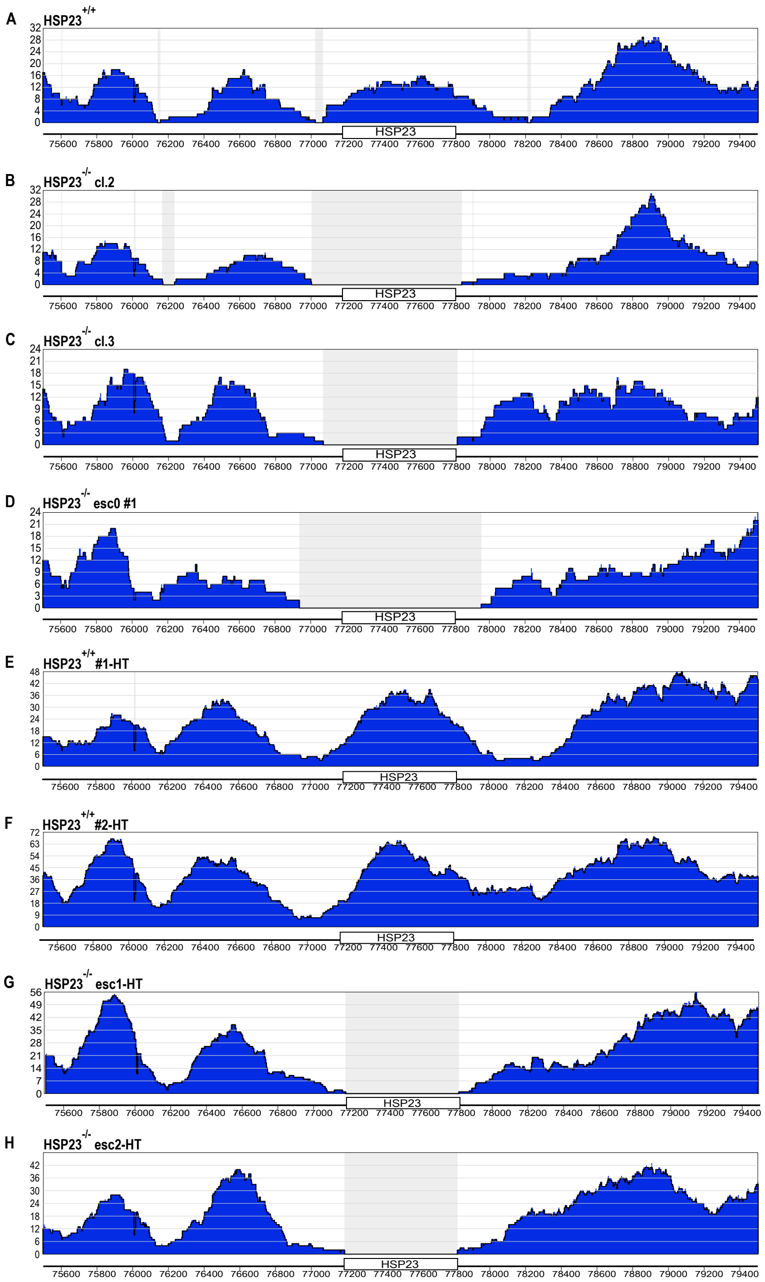

Fig S1 Verification of the *HSP23* gene replacement. Sequence reads from each analysed strain (A-H) were aligned to the reference DNA sequence consisting of chromosome 34 (TriTrypDB-46_LdonovaniBPK282A1_Genome.fasta). The Y-axis represents the number of reads and the X-axis shows the nucleotide position (bp) on chromosome 34. Grey shaded areas denote complete lack of aligned reads.

Supplementary Information

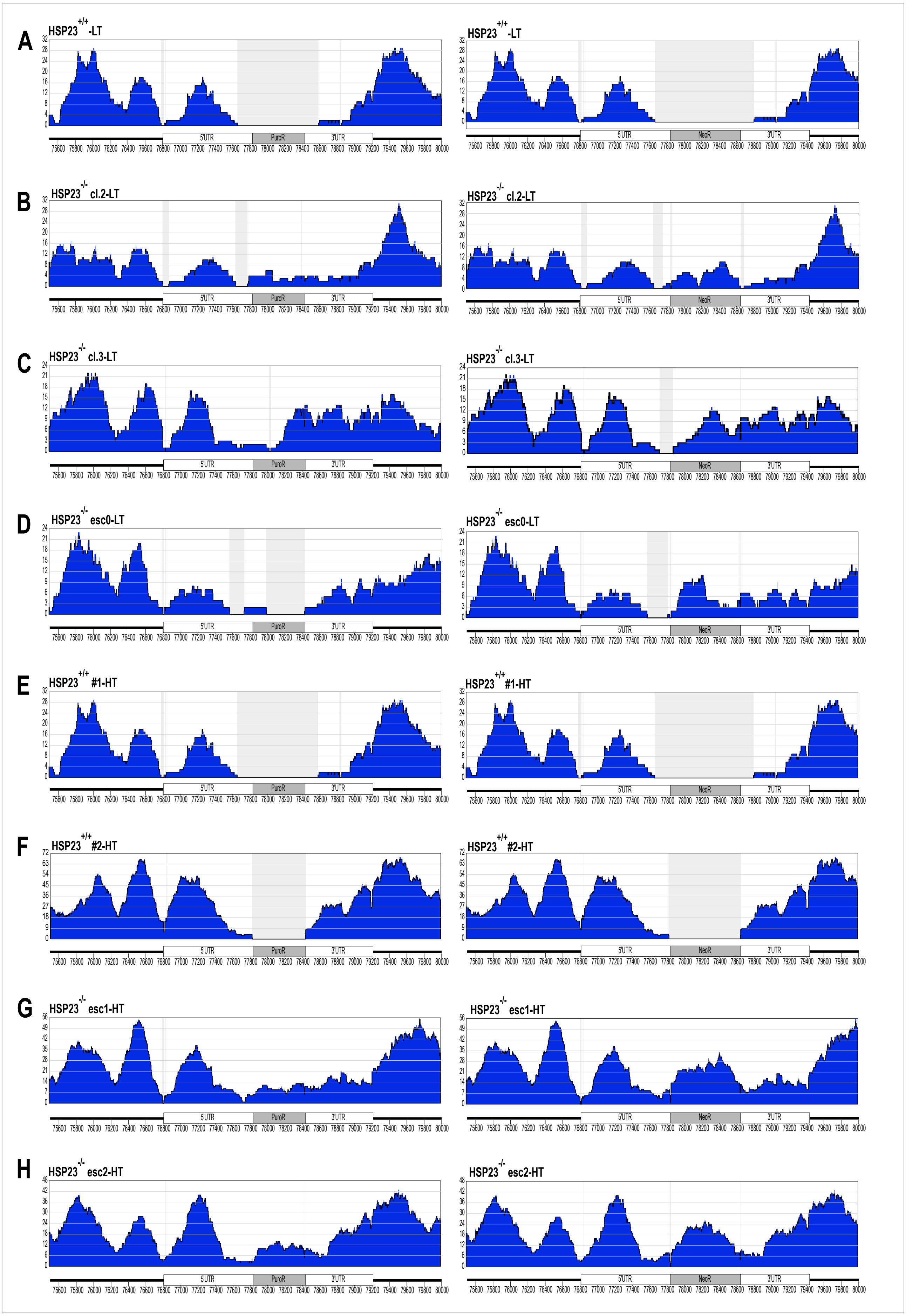

Fig S2 Verification of the *HSP23* gene replacement by the respective resistance cassettes. Sequence reads from each analysed strain (A-H) were aligned to the *in silico*-designed reference DNA sequences consisting of the *L. donovani* genome (DNA) sequence of chromosome 34 (TriTrypDB-46_LdonovaniBPK282A1_Genome.fasta) with the expected insertion of antibiotic resistance cassettes (NeoR = Neomycin; PuroR = Puromycin). The Y-axis represents the number of reads and the X-axis shows the nucleotide position (bp) on chromosome 34. Grey shaded areas denote complete lack of aligned reads.

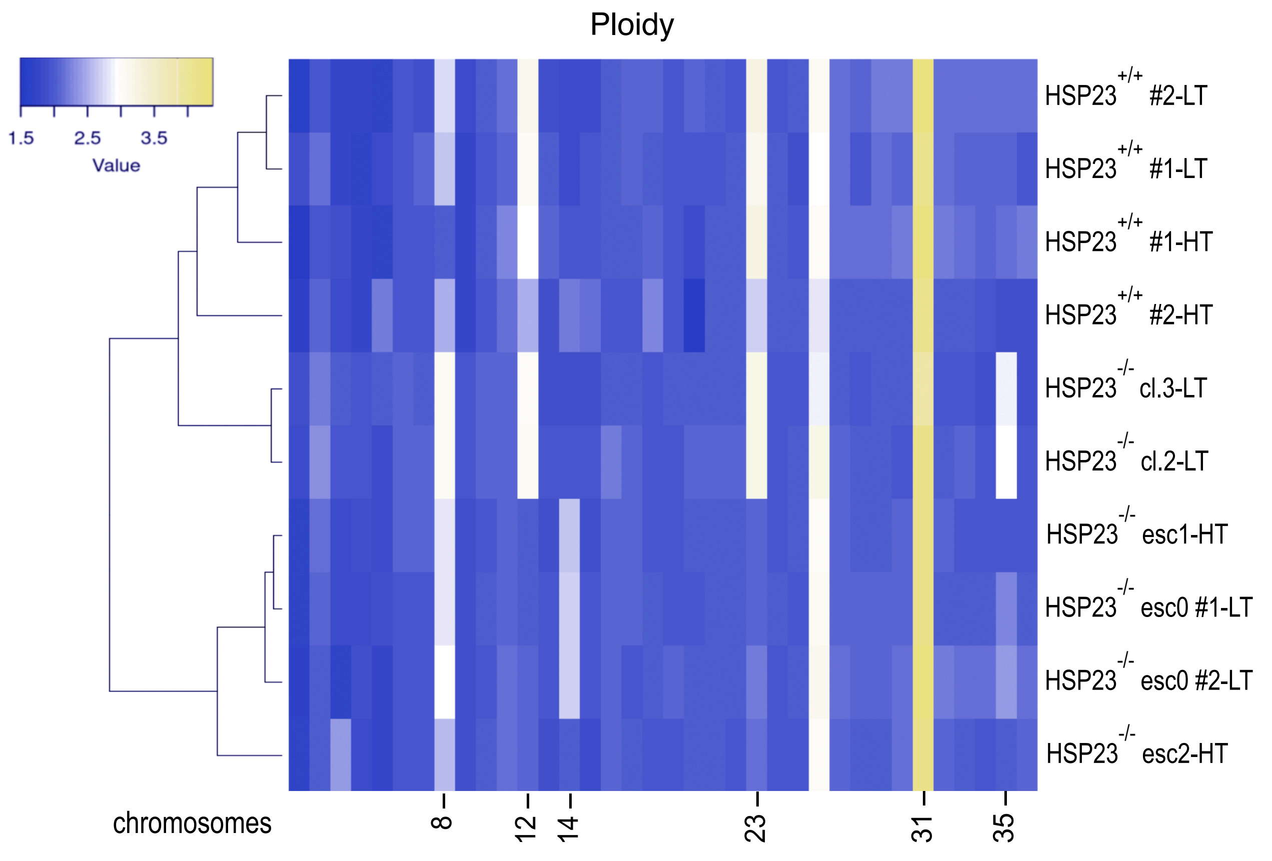

Fig S3: Dendrogram of chromosomal ploidy profiles. Heat map showing somy levels across all samples. The x-axis indicates individual chromosomes of *L. donovani*. Color scale indicates low (blue) to high (yellow) ploidy. The dendrogram shows clustering of the strains based on similarity of ploidy profiles and was generated by Pearson distance measurements.

Fig S4 Abundance of mRNA levels in transgenic over expression lines. mRNA was isolated from transgenic over expression lines HSP23$^{-/-}$ [Mix]-LT and HSP23$^{-/-}$ [Mix]-HT, selected at 25°C or 37°C, respectively. mRNA levels were determined for all genes of the six-gene cluster on chromosome 35 (LdBPK_351030-351080) and normalised to WT levels (set to 1).

A

strain #	HSP23	transgene(s)	NT sel.	temp. sel. at
1	+/+	-	-	LT
2	+/+	[CK1.2]	+	LT
3	-/- cl. 2	-	-	LT
4	-/- cl. 3	-	-	LT
5	-/- cl. 2	[CK1.2]	+	LT
6	-/- cl. 3	[CK1.2]	+	LT
9	-/- cl. 2	vector	+	LT

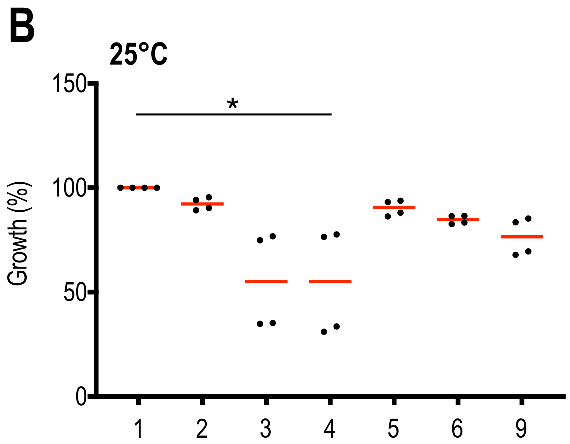

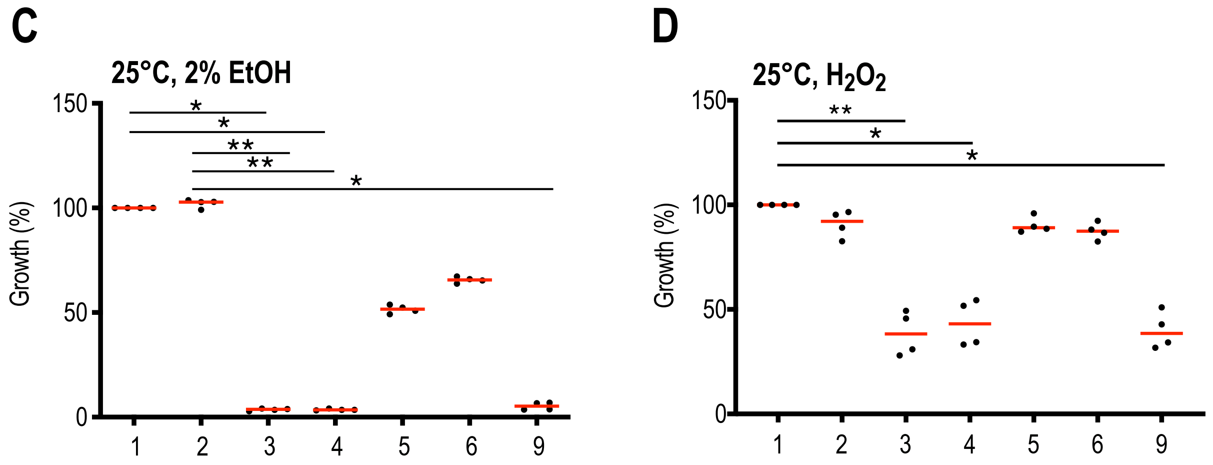

Fig S5 Characterisation of CK1.2 over expression lines. (A) List of transgenic parasite cell lines tested. NT sel = selected under nourseothricine; temp. sel. = temperature selection scheme. (B-D) 1×10^5 cells/ml were seeded into 7 ml of supplemented M199+ medium and grown for 4 days. Cell density on day 4 was then calculated as a percentage of wild-type ($HSP23^{+/+}$) cell density (set at 100%). Parasites were grown at 25˚C (B), 25°C and 2% EtOH (C), 25˚C and 200 nM H_2O_2 (D). Differences were tested for significance using the Kruskal–Wallis test: *=$p<0.05$, **=$p<0.01$ (n=4)

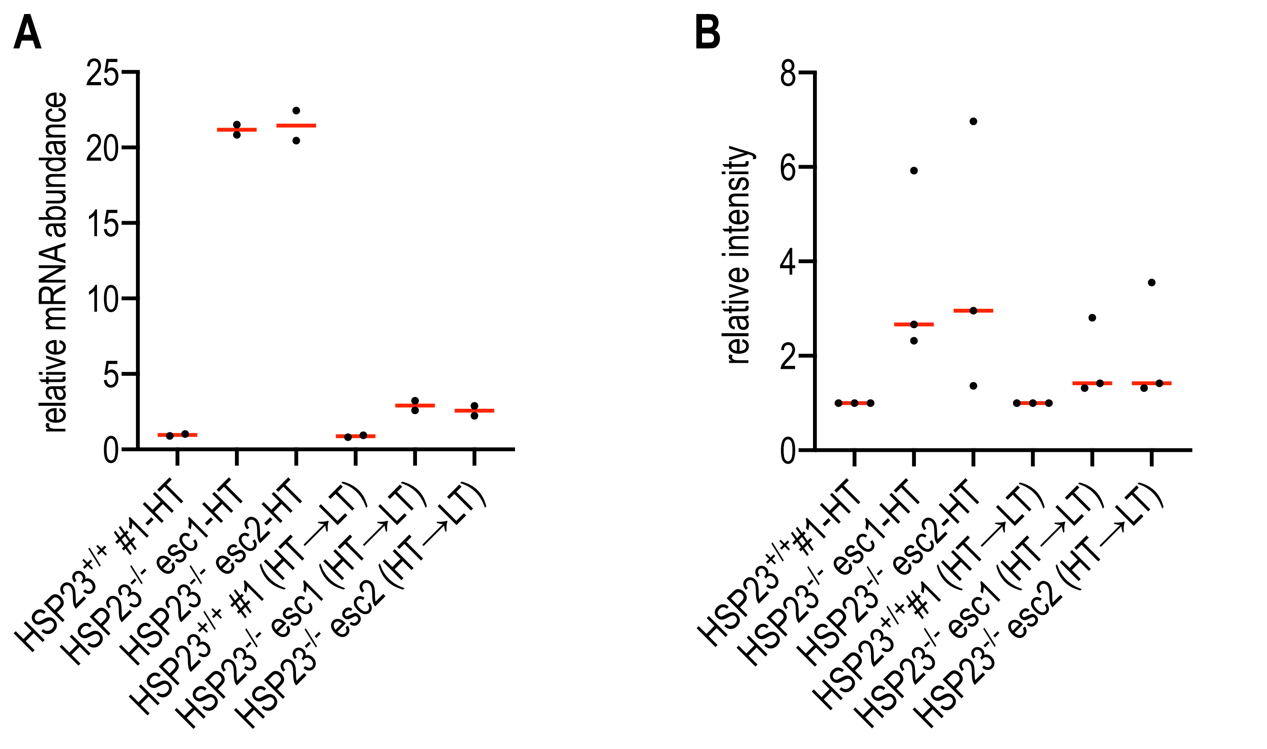

Fig S6 Abundance of CK1.2 in HSP23$^{-/-}$ esc1/2 mutants under different temperatures. CK1.2 mRNA (n=2) (A) and protein (n=3) (B) abundance were determined for HSP23$^{-/-}$ esc1/2 mutants that were selected first at HT (37°C) and were then transferred back to LT (25°C)(HT→LT). mRNA and protein samples were taken 30 days after transfer to LT conditions. For Western blot analysis, signal intensities were quantified using the Image J Software and were normalised to the WT (set to 1).

```
                                             CK1 =                                            CK1 =
                                             S/TXXS/T                                         S/TXXS/T
LdHsp23     1 MSTSGPLVPPISWAQRPEYVLITIPLQDTTGVSVEIKDEGRELC---FACSSPEHKQYACTIHFYGVISSEESQHVVRPRQIELKLRKKFSKSLEDANDDEVEWPRLTKE 107
LmjHsp23    1 MSSSGPLAPPISWAQRPEYVLITIPLQDTTGVSVEIKDEGRELH---FACSSPEHKQYACTIHFYGVISSEESQHVVRPRQIELKLRKKFSKSLEDANDDEVEWPRLTKE 107
LbrHsp23    1 MSASGSLVPPISWAQRPEYVLVTIPLQDTTGVTVEIKDEGRELL---FACCAPEGKQYACTIHFYGAISSEESQHVVRPRQIELKLRKKLTRSLEDA-DDEVEWPRLTKE 106
LmxHsp23    1 MSTSSPLAPPISWAQRPEYVLITIPLQDTTSVCVEIKDEGKELR---FACSSPEHKQYACTIHFYGAISSEESQHVVRPRQIELKLRKKFSKSLEDANDDEVEWPRLTKE 107
LdP23       1 MS-----HLPIKWAERKDRLFITVEASTPTDVQVNFQEKTVSISGNGITAKGSQPHALKDELHLLKEIVPEESTFKVLGMAIQICAIKK----------EKGYWNRLVDE  95

LdHsp23   108 KAKYPN--ISIDWSKWKDENDDECAADDLGDFGLSGGDAMDGQYSEMLSQLMQAQGQKDAEESAGLPPGTIPGFGSARGQEATHGACAVAGDDDGDMPPLEEDT--- 209
LmjHsp23  108 KSKYPN--ITIDWSKWKDENDDECAADDLGNFGLSGGDAMDGQYSEMLSQLMQAQGQKDAEESADLPPGTIPGFGSARGQEATHGASAETGDDDGDMPPLEEDT--- 209
LbrHsp23  107 KVKYPN--ITIDWSKWKDE-DDEGATDDLGDFGLGGGDAMDGQYSEMLSQLMQTQGKKDAEELAGLPPGTIPEFGSAQRQEATHGASAAAGDDDSEMPPLEDDM--- 207
LmxHsp23  108 KVKYPN--ITIDWSKWKDENDDECAADDLGDFGLSGGDAMDGQYSEMLSQLMQAQGQRDAEESAGLPTGTIPAFGSARGQEATHGTSAGAGDDDGDMPPLEEDT--- 209
LdP23      96 STKATKSWLSADWNLWKDEDDEAEEVAAASNFGGYG--DMGGMDMASMMGGMGGMGDMDMESMMAS-MGKDAGGDSDDEELDDDGAEMADGECTEEEPAADISDLNA 199
```

Fig S7 Protein sequence alignments of HSP23 orthologs in *Leishmania* spp. and *L. donovani* P23 protein. Putative CK1 phosphorylation sites (S/TXXS/T) are underlined and in bold font.

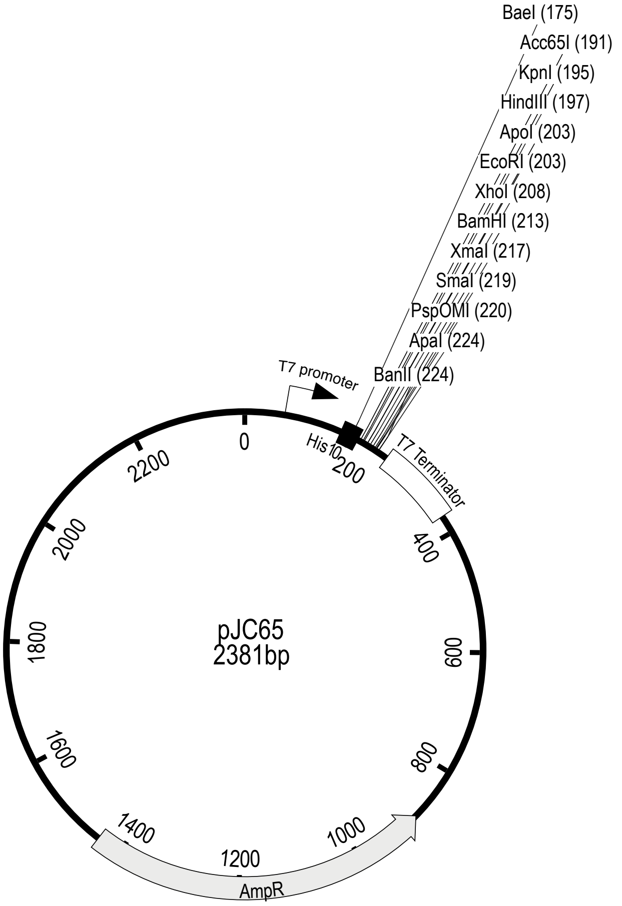

Fig S8 Schematic depiction plasmid used for protein expression in *E.coli*. pJC65 is derived from pJC45 (Schlüter et al., 2000), with a newly designed multiple cloning site.

Supplementary Information

Raw Images used for Figures

Quantification of CK1.2 protein levels shown in Fig 4A+Fig4B Repeat 1-4

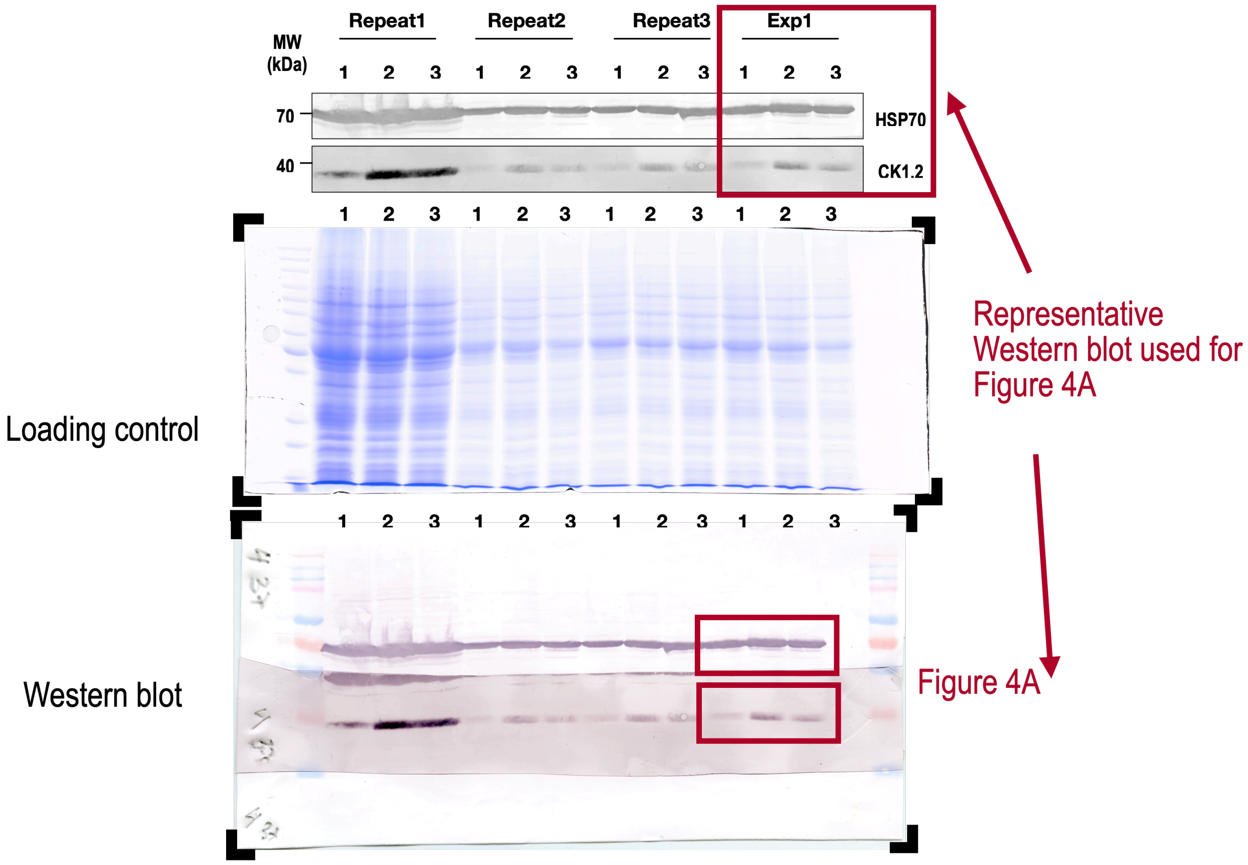

1: HSP23+/+-HT
2: HSP23-/- esc #1-HT
3: HSP23-/- esc #2-HT

Supplementary Information

Quantification of CK1.2 protein levels shown in Fig.4B Repeat4

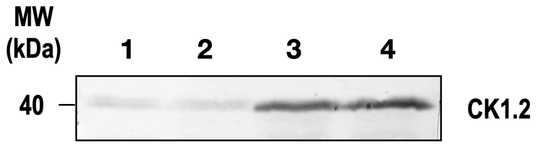

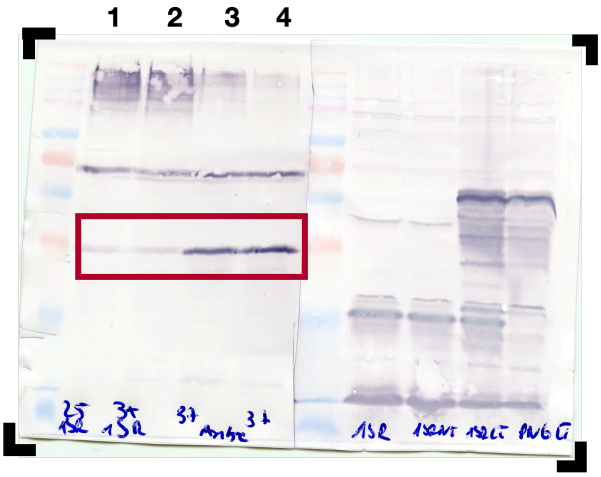

Western blot

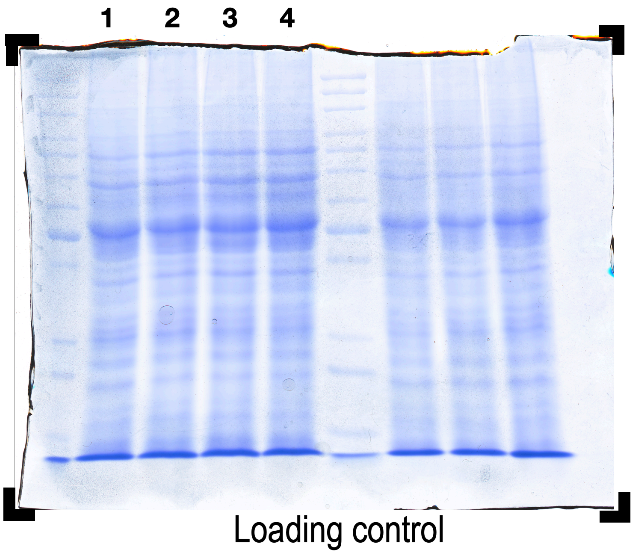

Loading control

1: HSP23+/+ -LT
2: HSP23+/+-HT
3: HSP23-/- esc #1-HT
4: HSP23-/- esc #2-HT

Quantification of CK1.2 protein levels shown in Fig 4B Repeat 5

Western blot

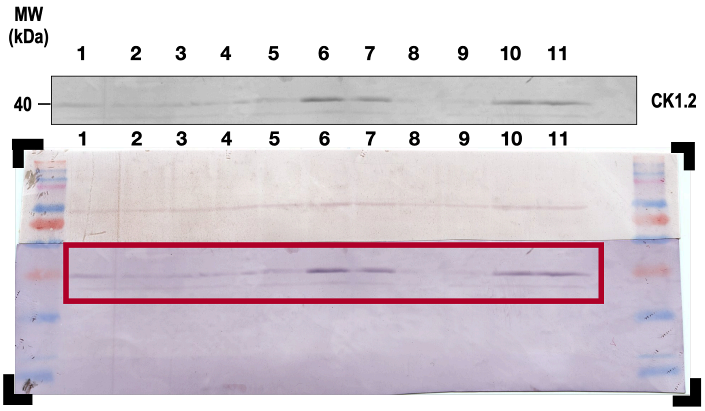

Loading control

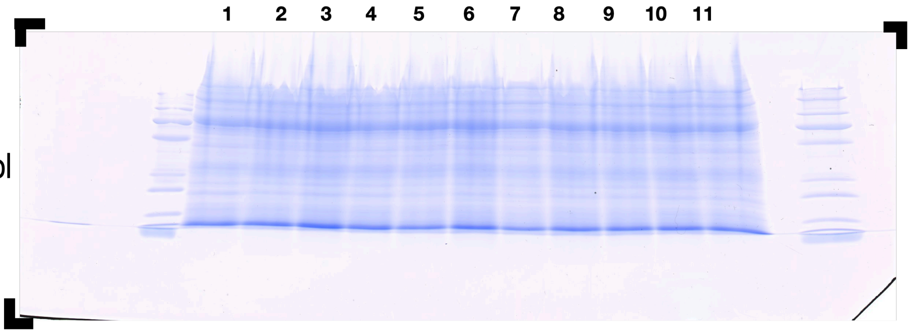

1: HSP23+/+-LT
2: HSP23-/- cl.2-LT
3: HSP23-/- cl.3-LT
4: HSP23-/- esc-LT
5: HSP23+/+-HT
6: HSP23-/- esc #1-HT
7: HSP23-/- esc #2-HT
8: HSP23-/- cl.2 [Mix]-LT
9: HSP23-/- cl.3 [Mix]-LT
10: HSP23-/- cl.2 [Mix]-HT
11: HSP23-/- cl.3 [Mix]-HT

Raw images Fig 5B (left panels)

1 2 3 M 1 2 3 M 1 2 3 4 5 6 **1 2 3 M 1 2 3 M 1 2 3 7 8 9**

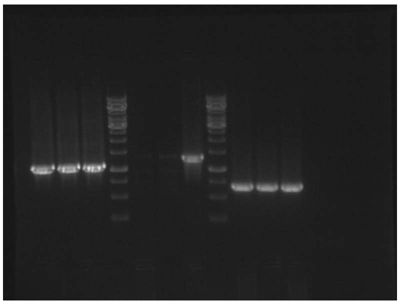

351030 351040 351050

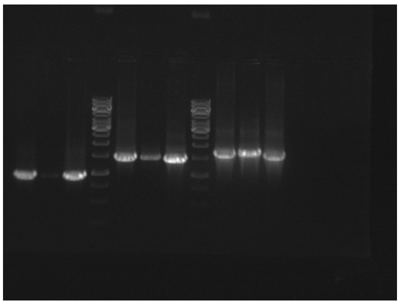

351060 351070 351080

Raw image Fig 5B (right panels)

10 11 12 M 10 11 12 M 10 11 12 **10 11 12 M 10 11 12 M 10 11 12**

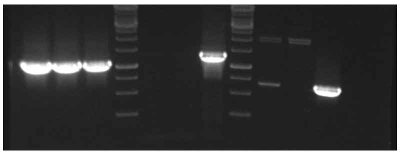

351030 351040 351050

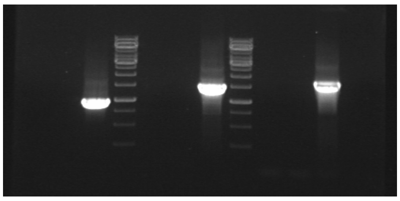

351060 351070 351080

1 : HSP23$^{-/-}$ cl.2 [Mix]-LT
2: HSP23$^{-/-}$ cl.3 [Mix]-LT
3: (+) ctrl
4: (-) ctrl for LdBPK_351030
5: (-) ctrl for LdBPK_351040
6: (-) ctrl for LdBPK_351050
7: (-) ctrl for LdBPK_351060
8: (-) ctrl for LdBPK_351070
9: (-) ctrl for LdBPK_351080
10: HSP23$^{-/-}$ cl.2 [Mix]-HT
11: HSP23$^{-/-}$ cl.3 [Mix]-HT
12: (+) ctrl
M : 1kb DNA ladder

Supplementary Information

Quantification of CK1.2 protein levels shown in Fig 6F cl.2 Repeat 1

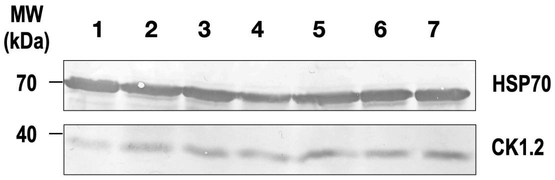

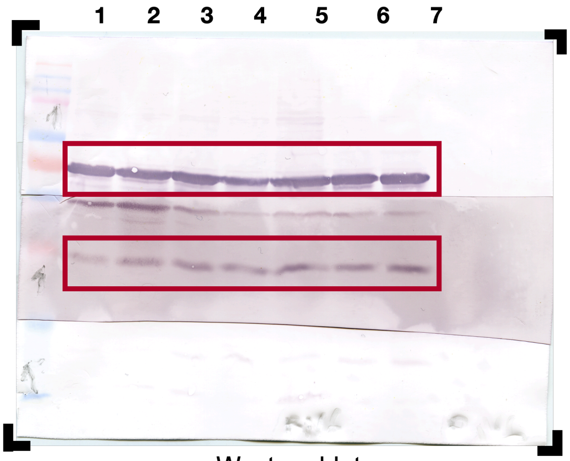

Western blot

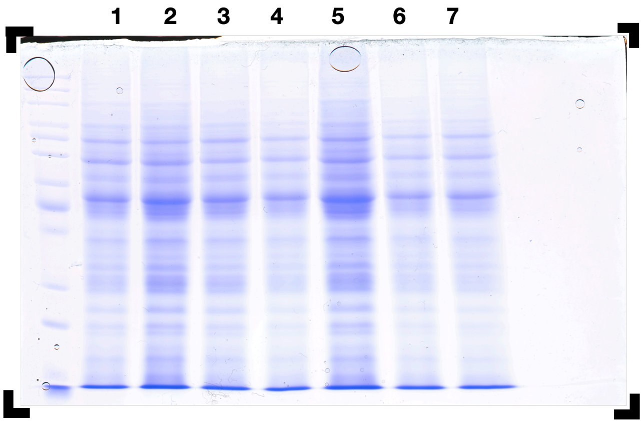

Loading control

1: HSP23+/+ -LT
2: HSP23-/- cl.2 [Mix]-LT
3: HSP23-/- cl.2 [Mix]-HT
4: HSP23-/- cl.2 [Mix]-LT—>HT
5: HSP23-/- cl.2 [CK1.2]-LT
6: HSP23-/- cl.2 [CK1.2]-HT
7: HSP23-/- cl.2 [CK1.2]-LT—> HT

Supplementary Information

Quantification of CK1.2 protein levels shown in Fig 6F cl.3 Repeat 1

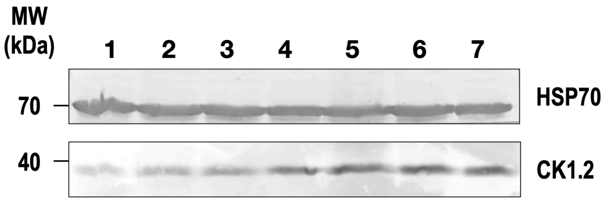

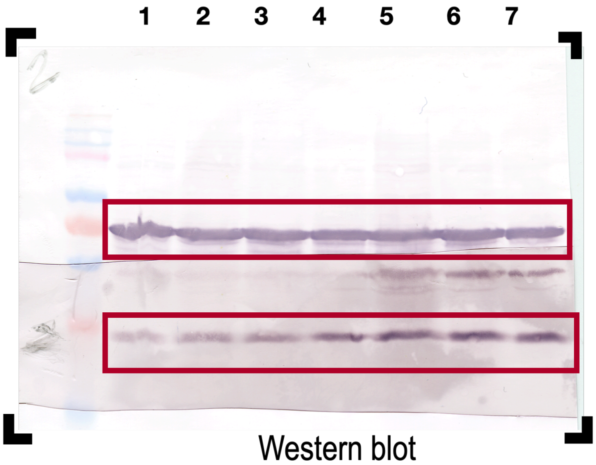

Western blot

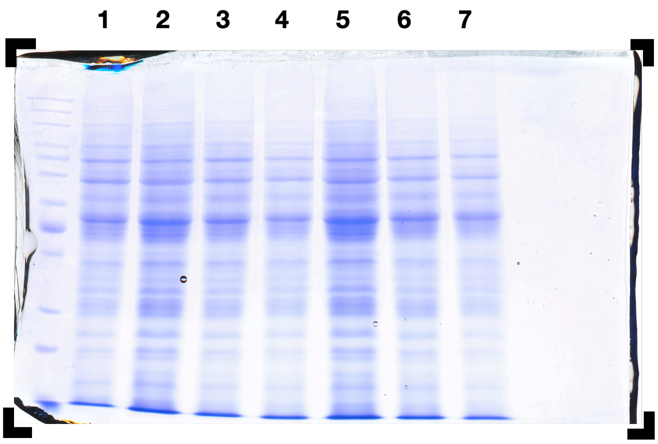

Loading control

1: HSP23+/+ -LT
2: HSP23-/- cl.3 [Mix]-LT
3: HSP23-/- cl.3 [Mix]-HT
4: HSP23-/- cl.3 [Mix]-LT—>HT
5: HSP23-/- cl.3 [CK1.2]-LT
6: HSP23-/- cl.3 [CK1.2]-HT
7: HSP23-/- cl.3 [CK1.2]-LT—> HT

Supplementary Information

Quantification of CK1.2 protein levels shown in Fig 6F cl.2 Repeat 2

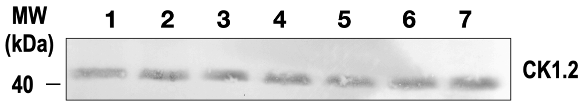

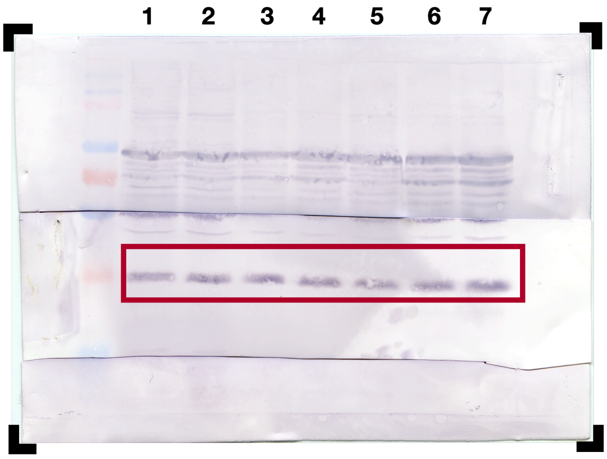

Western blot

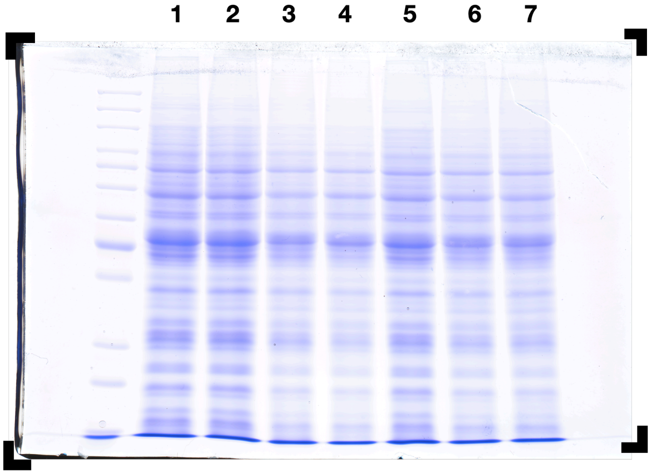

Loading control

1: HSP23+/+ -LT
2: HSP23-/- cl.2 [Mix]-LT
3: HSP23-/- cl.2 [Mix]-HT
4: HSP23-/- cl.2 [Mix]-LT—>HT
5: HSP23-/- cl.2 [CK1.2]-LT
6: HSP23-/- cl.2 [CK1.2]-HT
7: HSP23-/- cl.2 [CK1.2]-LT—> HT

Supplementary Information

Quantification of CK1.2 protein levels shown in Fig 6F cl.3 Repeat 2

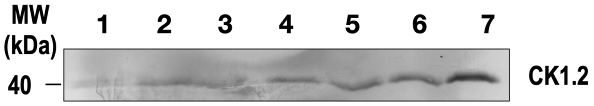

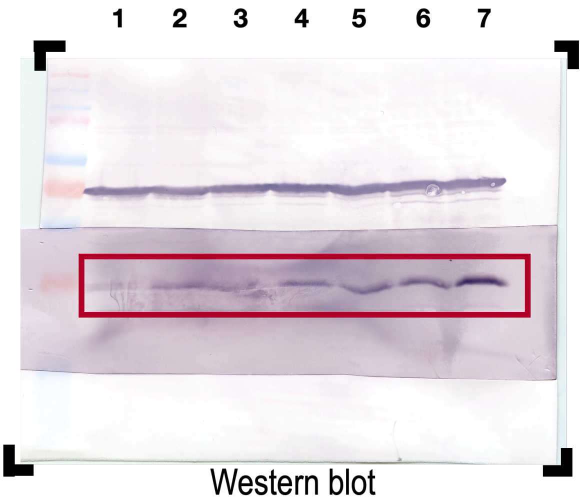

Western blot

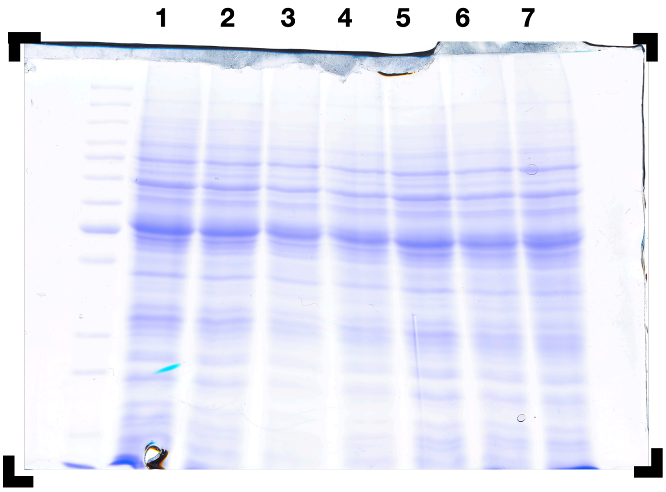

Loading control

1: HSP23+/+ -LT
2: HSP23-/- cl.3 [Mix]-LT
3: HSP23-/- cl.3 [Mix]-HT
4: HSP23-/- cl.3 [Mix]-LT—>HT
5: HSP23-/- cl.3 [CK1.2]-LT
6: HSP23-/- cl.3 [CK1.2]-HT
7: HSP23-/- cl.3 [CK1.2]-LT—> HT

Quantification of CK1.2 protein levels shown in Fig 6F cl.2+3 Repeat 3

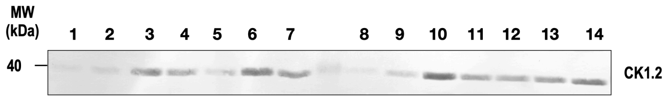

Western blot

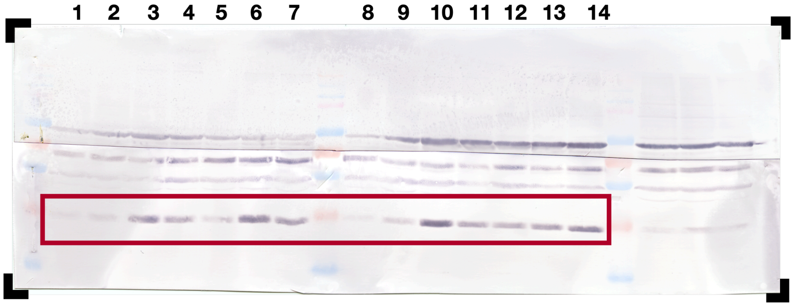

Loading control

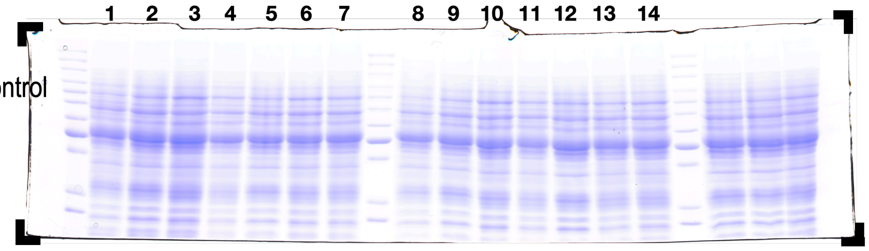

1: HSP23+/+ -LT
2: HSP23-/- cl.2 [Mix]-LT
3: HSP23-/- cl.2 [Mix]-HT
4: HSP23-/- cl.2 [Mix]-LT—>HT
5: HSP23-/- cl.2 [CK1.2]-LT
6: HSP23-/- cl.2 [CK1.2]-HT
7: HSP23-/- cl.2 [CK1.2]-LT—> HT
8: HSP23+/+ -LT
9: HSP23-/- cl.3 [Mix]-LT
10: HSP23-/- cl.3 [Mix]-HT
11: HSP23-/- cl.3 [Mix]-LT—>HT
12: HSP23-/- cl.3 [CK1.2]-LT
13: HSP23-/- cl.3 [CK1.2]-HT
14: HSP23-/- cl.3 [CK1.2]-LT—> HT

Representative Autoradiograph and Westernblot shown in Figure 7B: Repeat 1 HSP23

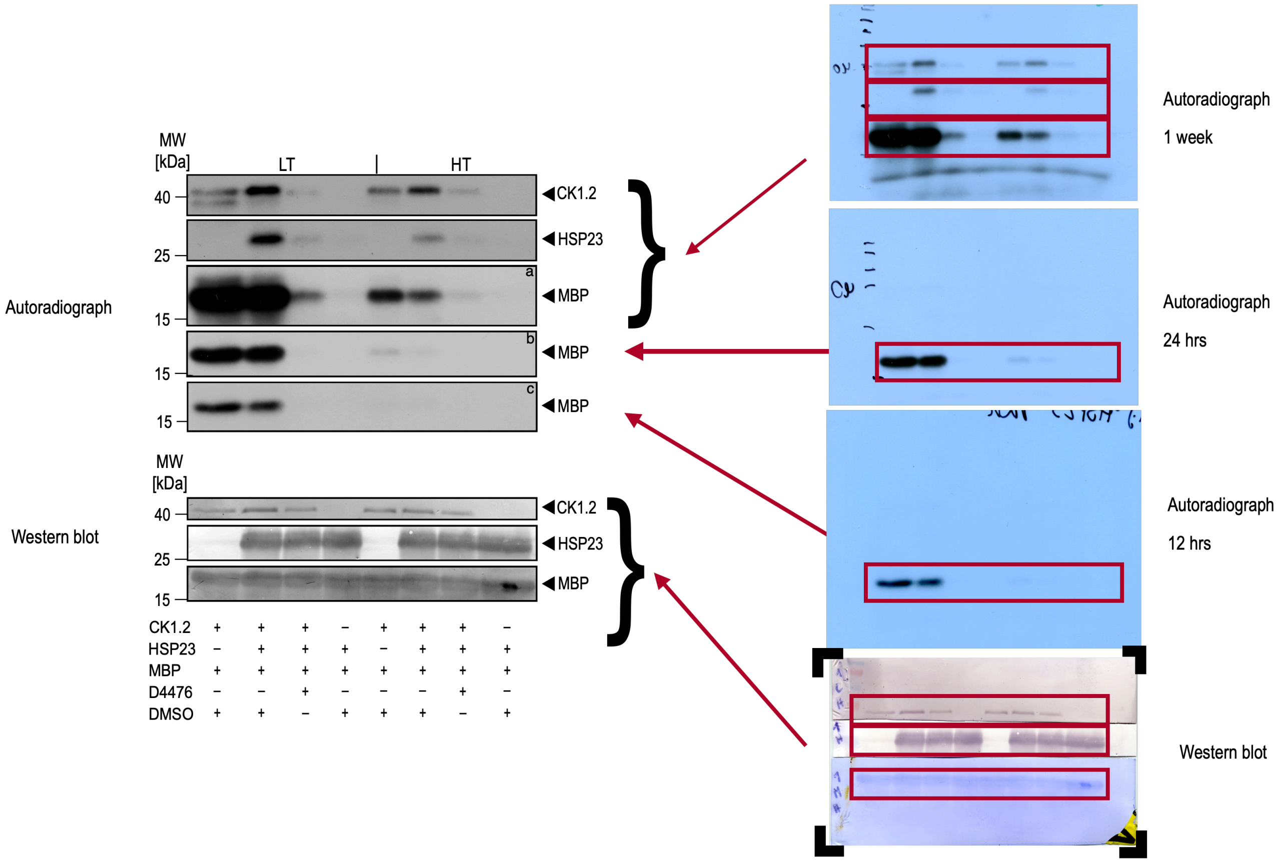

Supplementary Information

Additional data set for Fig.7: Repeat 2 HSP23

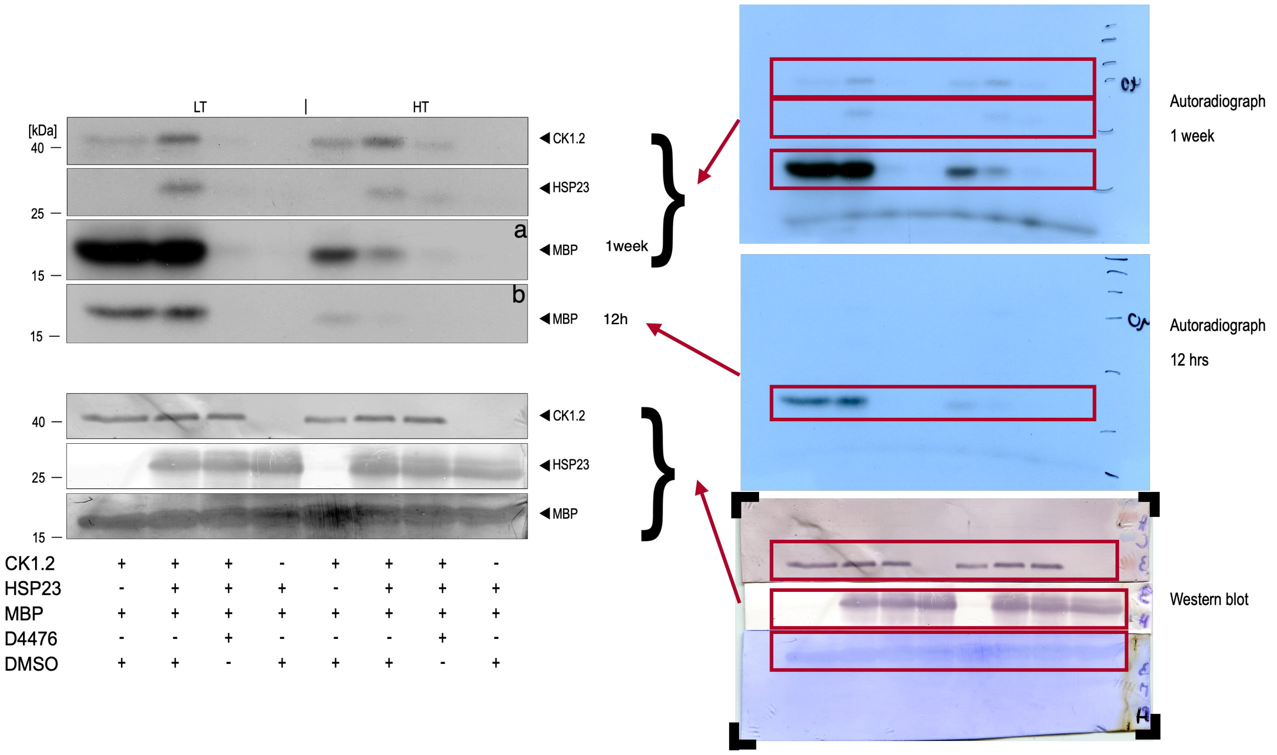

Supplementary Information

Additional data set for Fig.7: Repeat 3 HSP23

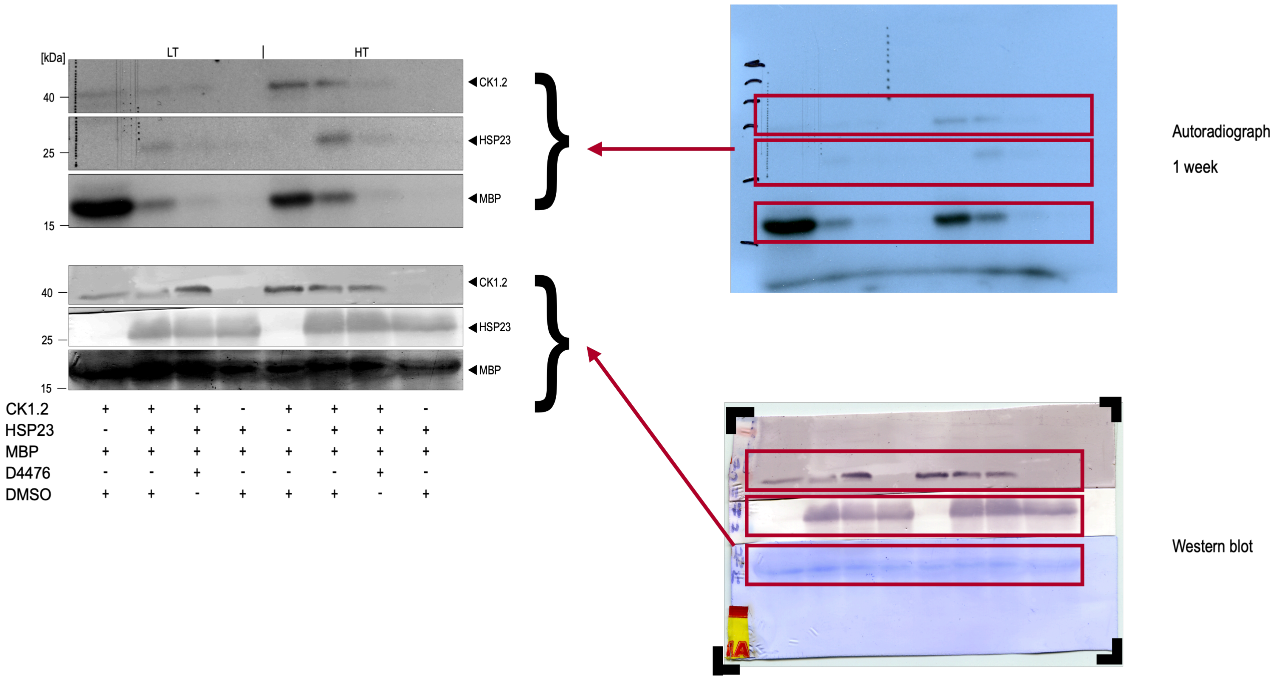

Additional data set for Fig.7: Repeat 4 HSP23

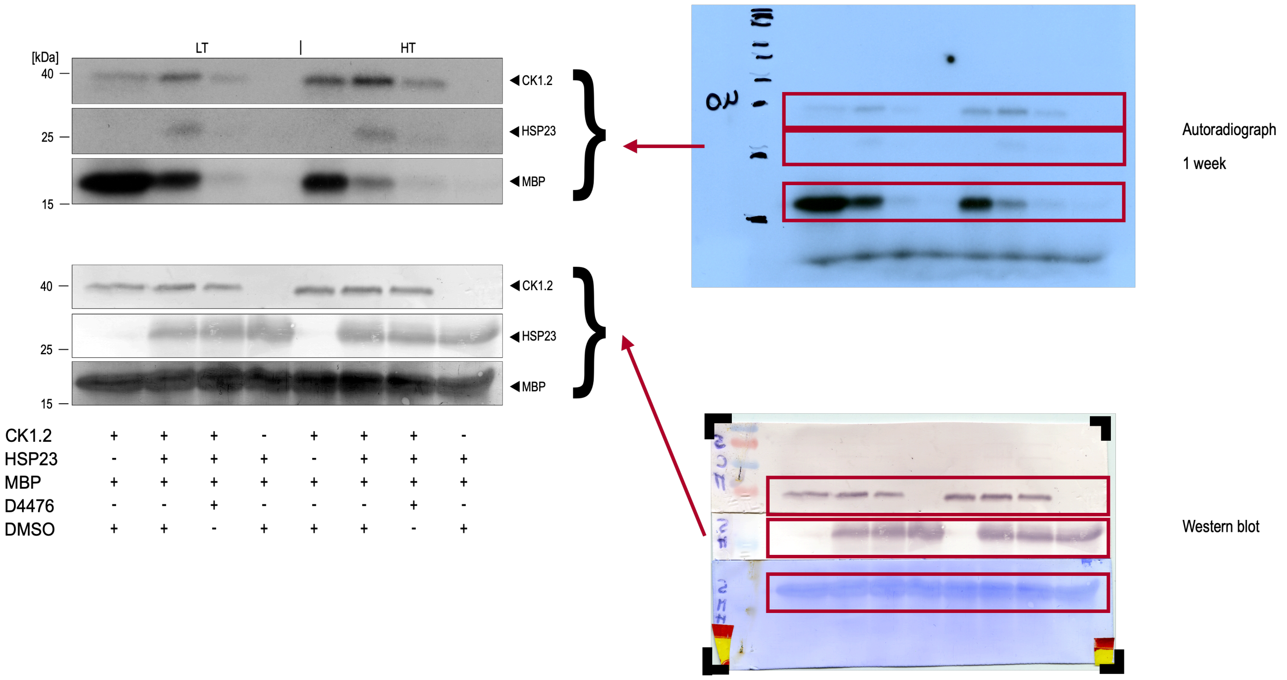

Representative Autoradiograph and Westernblot shown in Figure 7C: Repeat 1 p23

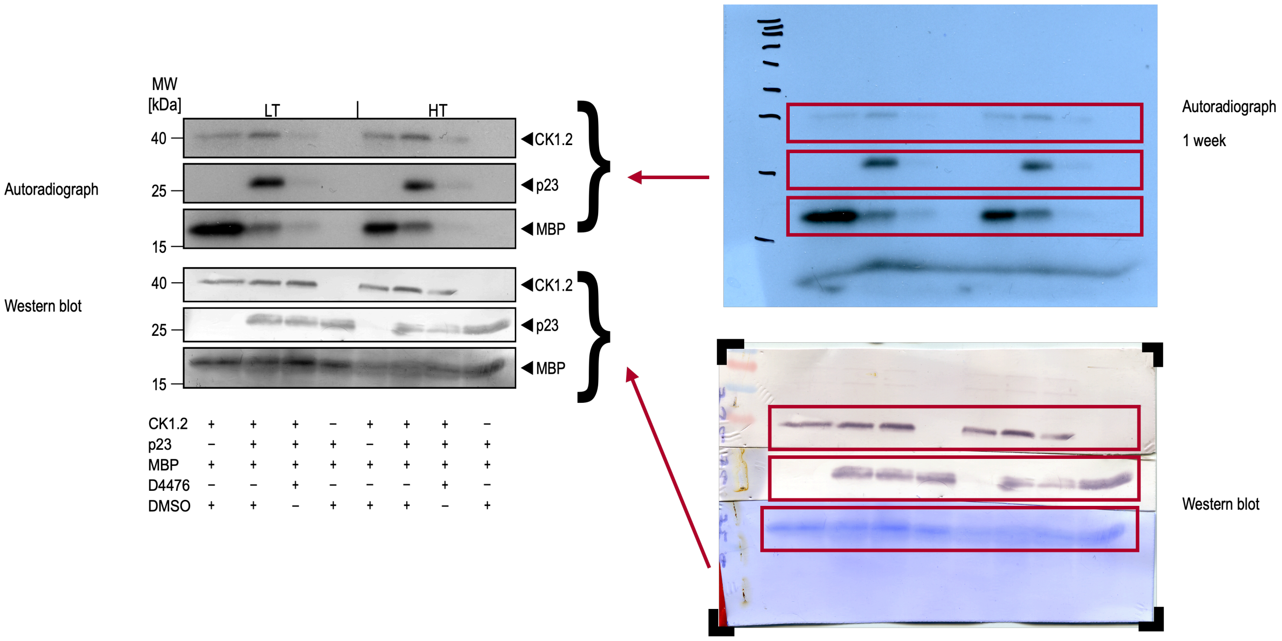

Supplementary Information

Additional data set for Fig.7:Repeat 2 p23

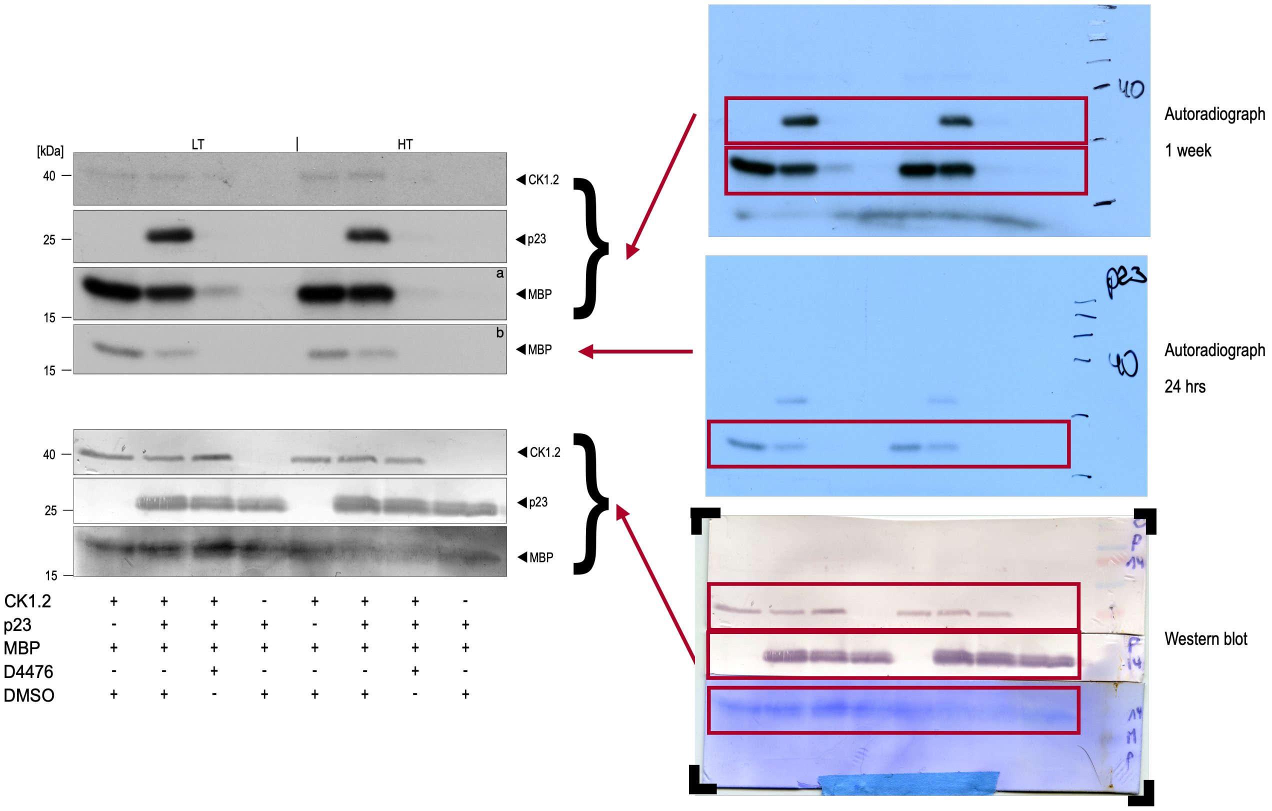

Supplementary Information

Additional data set for Fig.7: Repeat 3 p23

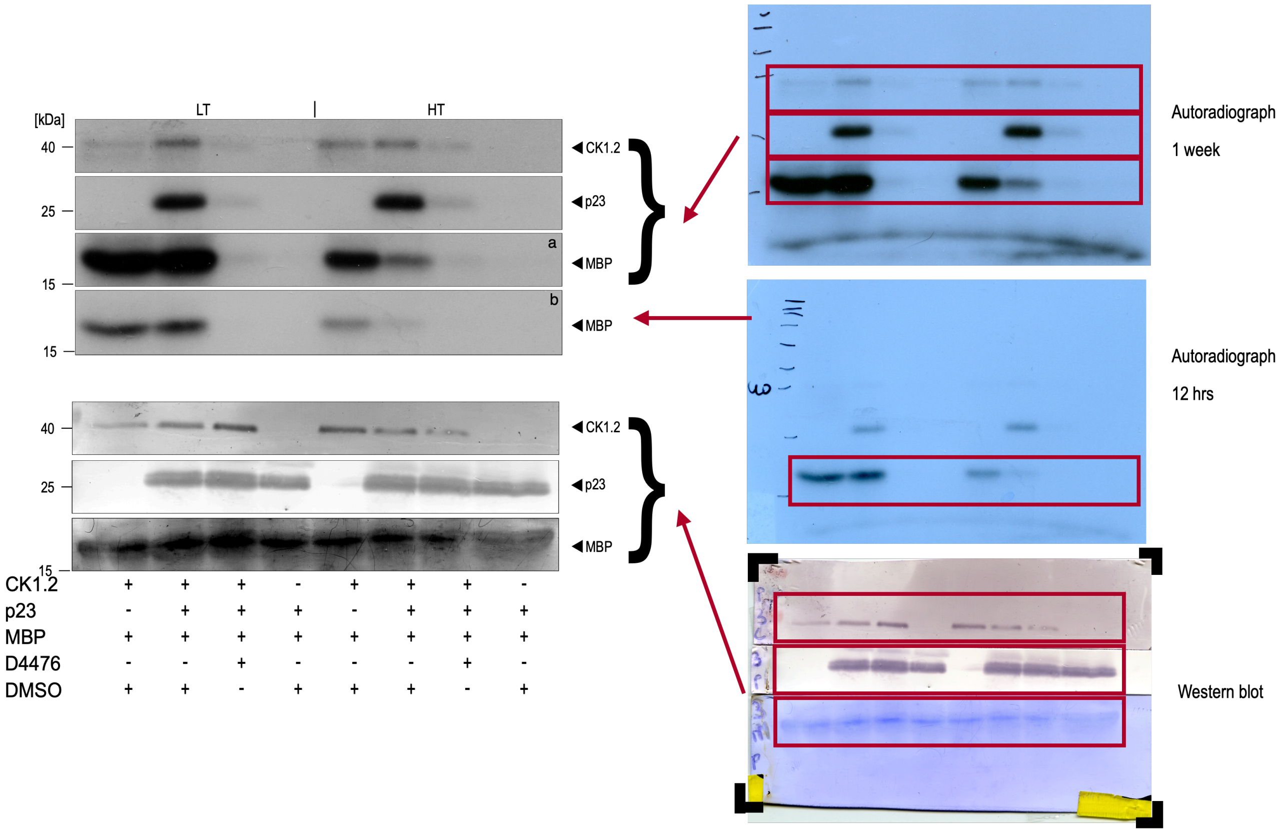

Additional data set for Fig.7: Repeat 4 p23

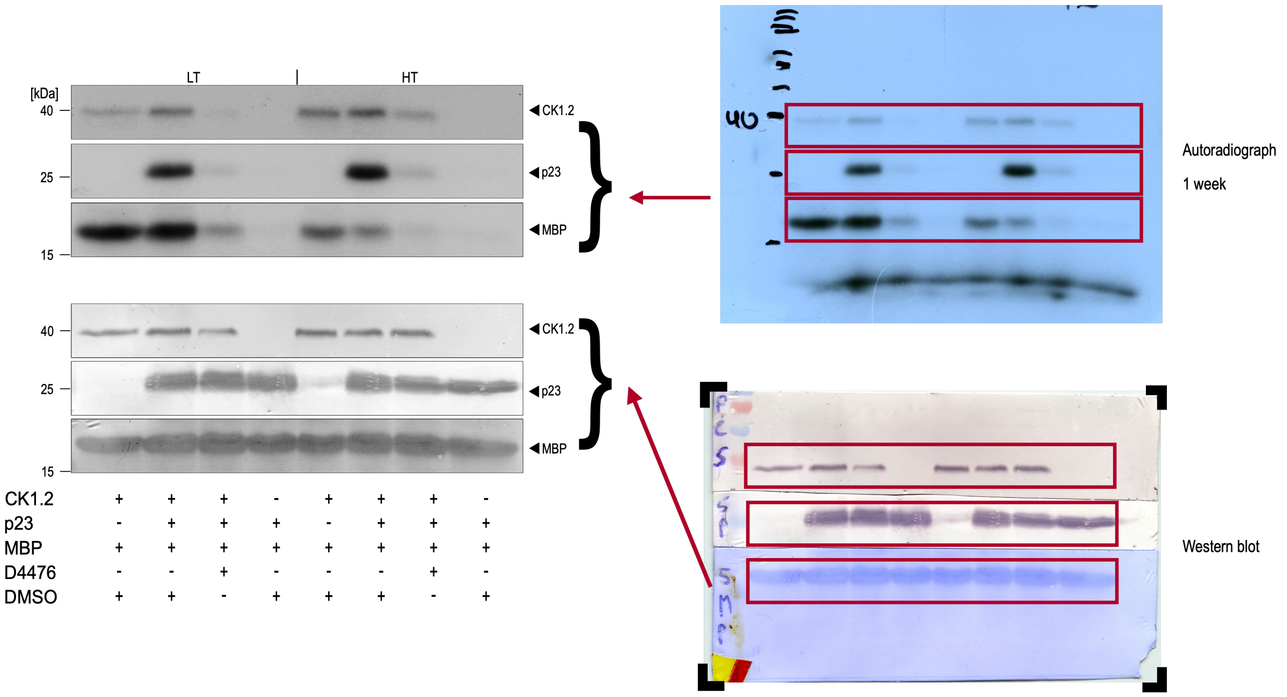

Quantification of CK1.2 protein level shown in Fig S6 Repeat 1

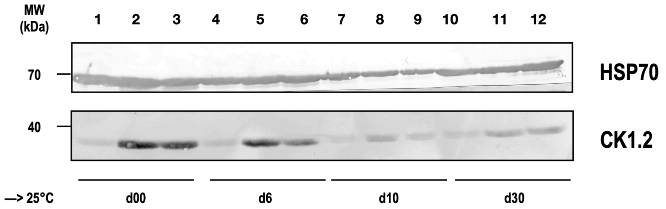

Loading control

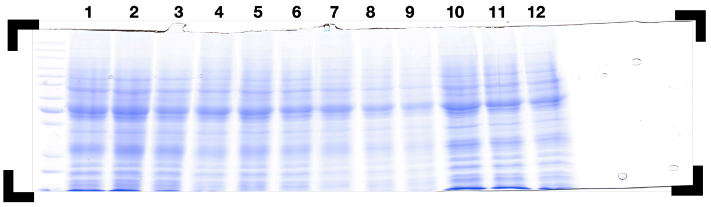

Western blot

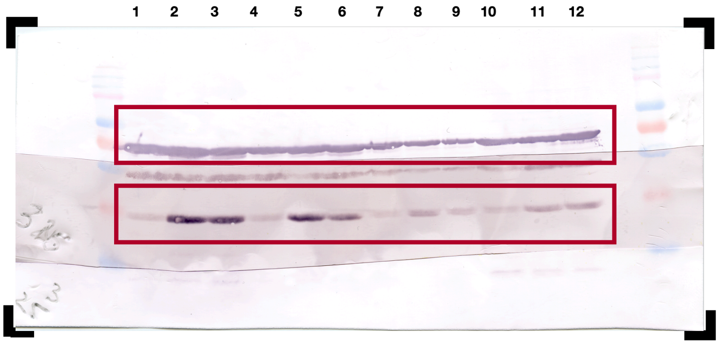

1: HSP23$^{+/+}$ -HT
2: HSP23$^{-/-}$ esc #1-HT
3: HSP23$^{-/-}$ esc #2-HT
4: HSP23$^{+/+}$ (HT→LT)
5: HSP23$^{-/-}$ esc #1 (HT→LT)
6: HSP23$^{-/-}$ esc #2 (HT→LT)
7: HSP23$^{+/+}$ (HT→LT)
8: HSP23$^{-/-}$ esc #1 (HT→LT)
9: HSP23$^{-/-}$ esc #2 (HT→LT)
10: HSP23$^{+/+}$ (HT→LT)
11: HSP23$^{-/-}$ esc #1 (HT→LT)
12: HSP23$^{-/-}$ esc #2 (HT→LT)

Quantification of CK1.2 protein level shown in Fig S6 Repeat 2+3

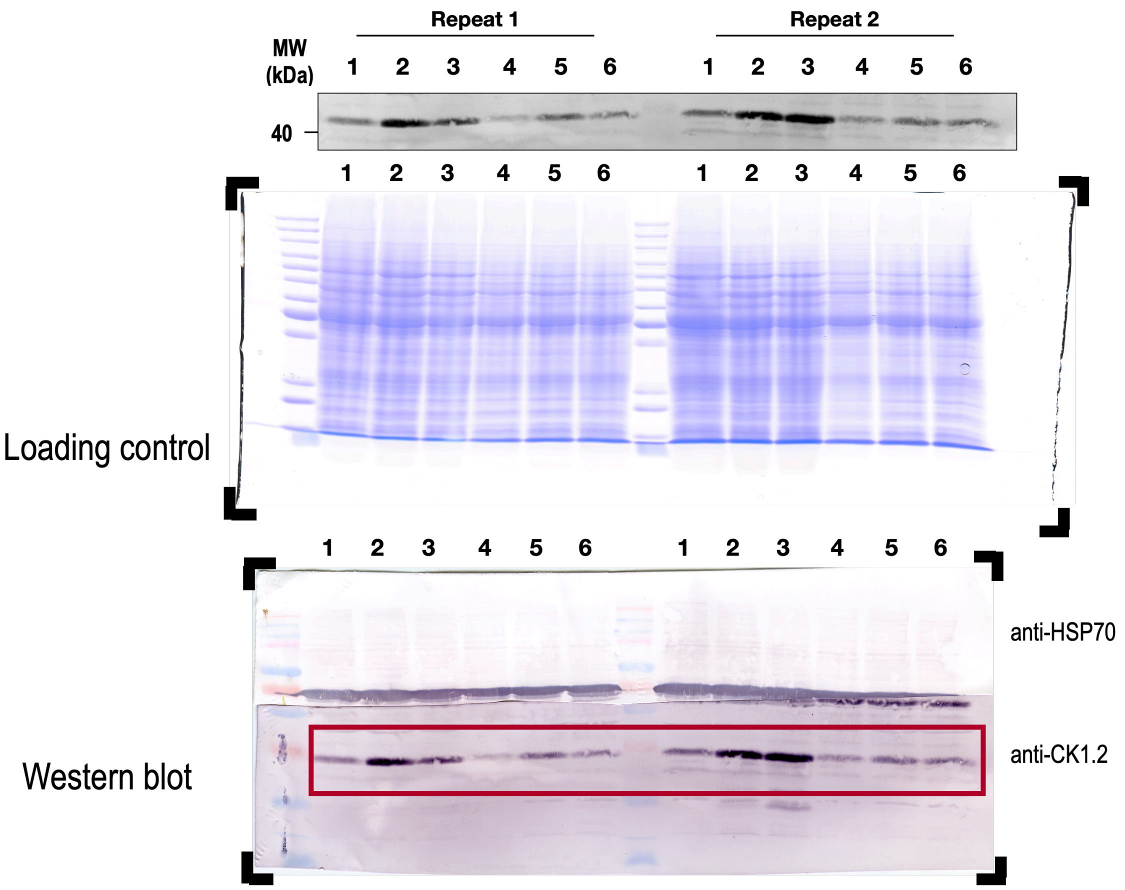

1: HSP23+/+-HT
2: HSP23-/- esc #1-HT
3: HSP23-/- esc #2-HT
4: HSP23+/+ (HT→LT)
5: HSP23-/- esc #1 (HT→LT)
6: HSP23-/- esc #2 (HT→LT)
