# Supplementary material for: Casein kinase 1.2 over expression restores stress resistance to Leishmania donovani HSP23 null mutants
Source: Sci Rep. 2020 Sep 29;10:15969. doi: 10.1038/s41598-020-72724-x (PMC7525241; doi:10.1038/s41598-020-72724-x)
Supplement: Supplementary file 2 — Supplementary Information 2. [file 41598_2020_72724_MOESM2_ESM.epub › OPS/cover.xhtml]

xml version="1.0" encoding="UTF-8"?
Supplementary Information

Supplementary Information

# 

# 

# Casein Kinase 1.2 Over Expression Restores Stress Resistance

to Leishmania donovani HSP23Null Mutants

Constanze Kröber-Boncardo1, Stephan Lorenzen2, Christine Brinker1, and Joachim Clos1\*

1Leishmania Group and 2Dept. of Epidemiology, Bernhard Nocht Institute for Tropical

Medicine, Hamburg, Germany

\* Correspondending author:

Joachim Clos, Bernhard Nocht Institute for Tropical Medicine, Bernhard Nocht St 74,

D-20359 Hamburg, Germany, email clos@bnitm.de, phone +49 40 42818 481
