# Supplementary material for: Casein kinase 1.2 over expression restores stress resistance to Leishmania donovani HSP23 null mutants
Source: Sci Rep. 2020 Sep 29;10:15969. doi: 10.1038/s41598-020-72724-x (PMC7525241; doi:10.1038/s41598-020-72724-x)
Supplement: Supplementary file 2 — Supplementary Information 2. [file 41598_2020_72724_MOESM2_ESM.epub › OPS/page-1.xhtml]

xml version="1.0" encoding="UTF-8"?
1 Page 1 | Supplementary Information

Supplementary Information

| Table S1 | |
| Primers used for CK1.2 study | Primer sequence 5’-3’ |
| qPCR |  |
| LdBPK\_340230.1\_fwd | CCATTCCTGGATTTGGCTCGG |
| LdBPK\_340230.1\_rev | GCATATCGCCGTCGTCATCTCC |
| LdBPK\_351030.1\_fwd | CACGTGTACATCATCGACTTTGG |
| LdBPK\_351030.1\_rev | CTCTTGCCTTCCTTGTATGGGAT |
| LdBPK\_351040.1\_fwd | ACACTCTCCCTTCGCATTATCAG |
| LdBPK\_351040.1\_rev | CTCGATGAAGTAGATCGGTTGCT |
| LdBPK\_351050.1\_fwd | TTTTGGATTAAGCGCCTGCAAC |
| LdBPK\_351050.1\_rev | GAAAGTAAATGGTCGTGTAGGCG |
| LdBPK\_351060.1\_fwd | CAGTACTACACACACAACCCGTA |
| LdBPK\_351060.1\_rev | CGACAACTTCTTGGTAGACTGGA |
| LdBPK\_351070.1\_fwd | TAGTCTTCTACGTGCTTCTCTGC |
| LdBPK\_351070.1\_rev | GTGATTTGGGTTGAAGAGGACAC |
| LdBPK\_351080.1\_fwd | CGAGTACGGCAACTTCAACGATA |
| LdBPK\_351080.1\_rev | CCGGTCGTCTATGTCATCTTCTT |
| over expression plasmids  construction |  |
| 351030\_KpnI\_fwd | GGAGGGTACCATGAACGTGGAACTGCGCGTC |
| 351030\_NdeI\_rev | GGAGCATATGCTACTGCTGTTCTTGCGCAC |
| 351040\_KpnI\_fwd | GGAGGGTACCATGCACACGCCACCCCTCTC |
| 351040\_NdeI\_rev | GGAGCATATGTCACATGAAGCGAGCCGCCA |
| 351050\_KpnI\_fwd | GGAGGGTACCATGACCTCCCCGGCAGCCGC |
| 351050\_NdeI\_rev | GGAGCATATGCTAGATTCCTGTTGTCTGTAAG |
| 351060\_NdeI\_fwd | GGAGCATATGATGACGAGCCAGTCGCACGTG |
| 351060\_BglII\_rev | GGAGAGATCTTTAGGGCTCCATCACCATCG |
| 351070\_KpnI\_fwd | GGAGGGTACCATGACCGTTCTGCCACCCAAG |
| 351070\_NdeI\_rev | GGAGCATATGTCACTTGCGCAAAGCGTCGG |
| 351080\_KpnI\_fwd | GGAGGGTACCATGGGTAAGCGAAACGAAAG |
| 351080\_NdeI\_rev | GGAGCATATGCTACATGGATGCGACCACGC |
| pJC65 multiple cloning site |  |
| Link-pJC65+ | CATGGGCCATCATCATCATCATCATCATCATCATCACGGTGGTACCA |
| Link-pJC65- | AGCTTGGTACCACCGTGATGATGATGATGATGATGATGATGATGGCC |
