# Supplementary material for: Casein kinase 1.2 over expression restores stress resistance to Leishmania donovani HSP23 null mutants
Source: Sci Rep. 2020 Sep 29;10:15969. doi: 10.1038/s41598-020-72724-x (PMC7525241; doi:10.1038/s41598-020-72724-x)
Supplement: Supplementary file 2 — Supplementary Information 2. [file 41598_2020_72724_MOESM2_ESM.epub › OPS/page-2.xhtml]

xml version="1.0" encoding="UTF-8"?
2 Page 2 | Supplementary Information

Supplementary Information

| Table S2: Ploidy values for chromosomes based on NGS sequence read coverages. | | | | | | | | | | |
| CHR | HSP23+/+  #1-LT | HSP23+/+  #2-LT | HSP23+/+  #1-HT | HSP23+/+  #2-HT | HSP23-/-  cl.2-LT | HSP23-/-  cl.3-LT | HSP23-/-  esc #1-LT | HSP23-/-  esc #2-LT | HSP23-/-  esc #1-HT | HSP23-/-  esc #2-HT |
| 1 | 1.91 | 1.61 | 1.56 | 1.63 | 1.85 | 1.89 | 1.66 | 1.70 | 1.74 | 1.69 |
| 2 | 2.12 | 1.95 | 1.96 | 2.06 | 2.35 | 2.20 | 2.03 | 2.09 | 2.13 | 2.00 |
| 3 | 1.80 | 1.78 | 1.87 | 1.81 | 1.97 | 2.02 | 1.69 | 1.84 | 1.86 | 2.37 |
| 4 | 1.70 | 1.79 | 1.80 | 1.80 | 1.98 | 1.93 | 1.87 | 1.85 | 1.87 | 1.83 |
| 5 | 1.82 | 1.74 | 1.73 | 2.22 | 1.86 | 2.02 | 1.77 | 1.87 | 1.85 | 1.78 |
| 6 | 1.95 | 1.95 | 1.94 | 1.96 | 2.06 | 2.10 | 1.93 | 1.98 | 2.06 | 1.95 |
| 7 | 2.07 | 1.92 | 1.95 | 1.93 | 2.08 | 2.00 | 1.98 | 1.98 | 2.05 | 1.96 |
| 8 | 2.66 | 2.78 | 2.00 | 2.48 | 3.06 | 3.07 | 2.92 | 2.84 | 2.79 | 2.55 |
| 9 | 1.77 | 1.86 | 1.80 | 1.76 | 1.97 | 1.94 | 1.88 | 1.92 | 1.88 | 1.87 |
| 10 | 1.94 | 1.99 | 2.01 | 1.97 | 2.07 | 2.08 | 1.96 | 1.99 | 1.97 | 1.99 |
| 11 | 2.09 | 2.14 | 2.26 | 2.12 | 2.05 | 2.09 | 2.15 | 2.05 | 2.08 | 2.12 |
| 12 | 3.04 | 3.11 | 2.92 | 2.51 | 3.03 | 3.01 | 2.05 | 1.99 | 2.01 | 2.05 |
| 13 | 2.02 | 1.90 | 2.10 | 1.90 | 1.97 | 1.91 | 1.96 | 1.96 | 1.92 | 1.88 |
| 14 | 1.82 | 1.86 | 1.95 | 2.22 | 1.97 | 1.91 | 2.71 | 2.71 | 2.66 | 2.02 |
| 15 | 1.94 | 1.84 | 1.97 | 2.17 | 1.96 | 1.91 | 1.98 | 1.97 | 1.92 | 1.86 |
| 16 | 1.99 | 2.01 | 2.04 | 1.97 | 2.19 | 2.03 | 2.07 | 2.10 | 2.07 | 2.08 |
| 17 | 2.10 | 2.05 | 1.99 | 1.96 | 2.09 | 2.02 | 1.98 | 2.05 | 2.05 | 1.99 |
| 18 | 2.02 | 2.07 | 2.11 | 2.29 | 1.98 | 1.93 | 1.99 | 2.00 | 1.96 | 1.98 |
| 19 | 1.93 | 1.98 | 1.96 | 1.96 | 1.97 | 2.00 | 2.06 | 1.97 | 1.96 | 1.93 |
| 20 | 1.95 | 2.06 | 1.84 | 1.50 | 2.08 | 2.02 | 2.00 | 1.96 | 2.01 | 2.04 |
| 21 | 1.98 | 1.99 | 2.04 | 2.01 | 2.07 | 2.01 | 1.99 | 2.02 | 2.02 | 2.00 |
| 22 | 2.01 | 1.98 | 1.99 | 2.01 | 2.06 | 2.01 | 2.00 | 2.00 | 1.99 | 1.95 |
| 23 | 3.11 | 3.22 | 3.25 | 2.67 | 3.15 | 3.16 | 2.18 | 2.08 | 2.11 | 2.14 |
| 24 | 2.01 | 1.96 | 2.00 | 2.01 | 1.98 | 1.93 | 1.98 | 2.00 | 1.98 | 1.98 |
| 25 | 1.92 | 1.99 | 1.98 | 2.00 | 1.99 | 1.94 | 1.99 | 1.97 | 2.01 | 1.97 |
| 26 | 2.95 | 3.05 | 2.98 | 2.82 | 3.15 | 2.87 | 3.11 | 3.03 | 3.02 | 3.01 |
| 27 | 2.14 | 2.16 | 2.17 | 2.00 | 2.07 | 2.01 | 2.17 | 2.06 | 2.05 | 2.07 |
| 28 | 1.96 | 2.11 | 2.17 | 2.00 | 2.00 | 1.94 | 2.10 | 2.05 | 2.04 | 2.03 |
| 29 | 2.17 | 2.18 | 2.12 | 2.00 | 1.99 | 2.01 | 2.09 | 2.05 | 2.01 | 2.04 |
| 30 | 2.10 | 2.20 | 2.19 | 2.01 | 1.98 | 2.00 | 2.17 | 2.05 | 2.05 | 2.12 |
| 31 | 4.11 | 4.30 | 4.37 | 4.07 | 4.16 | 3.88 | 4.32 | 4.16 | 4.17 | 4.18 |
| 32 | 2.12 | 2.17 | 2.18 | 2.02 | 2.00 | 1.94 | 2.18 | 2.02 | 2.09 | 2.08 |
| 33 | 2.09 | 2.16 | 2.17 | 2.03 | 2.06 | 1.94 | 2.17 | 2.00 | 1.97 | 2.01 |
| 34 | 2.09 | 2.12 | 2.11 | 1.96 | 1.98 | 1.92 | 2.16 | 2.00 | 1.94 | 1.97 |
| 35 | 2.11 | 2.13 | 2.13 | 1.91 | 2.93 | 2.87 | 2.36 | 2.26 | 1.98 | 2.02 |
| 36 | 1.95 | 2.16 | 2.19 | 1.91 | 1.97 | 1.91 | 2.16 | 2.00 | 1.98 | 2.05 |
