# Supplementary material for: Casein kinase 1.2 over expression restores stress resistance to Leishmania donovani HSP23 null mutants
Source: Sci Rep. 2020 Sep 29;10:15969. doi: 10.1038/s41598-020-72724-x (PMC7525241; doi:10.1038/s41598-020-72724-x)
Supplement: Supplementary file 2 — Supplementary Information 2. [file 41598_2020_72724_MOESM2_ESM.epub › OPS/page-3.xhtml]

xml version="1.0" encoding="UTF-8"?
3 Page 3 | Supplementary Information

Supplementary Information

|  |
| Fig S1 Verification of the HSP23gene replacement. Sequence reads from each analysed strain  (A-H) were aligned to the reference DNA sequence consisting of chromosome 34  (TriTrypDB-46\_LdonovaniBPK282A1\_Genome.fasta). The Y-axis represents the number of reads  and the X-axis shows the nucleotide position (bp) on chromosome 34. Grey shaded areas denote  complete lack of aligned reads. |
