# Supplementary material for: Casein kinase 1.2 over expression restores stress resistance to Leishmania donovani HSP23 null mutants
Source: Sci Rep. 2020 Sep 29;10:15969. doi: 10.1038/s41598-020-72724-x (PMC7525241; doi:10.1038/s41598-020-72724-x)
Supplement: Supplementary file 2 — Supplementary Information 2. [file 41598_2020_72724_MOESM2_ESM.epub › OPS/page-5.xhtml]

xml version="1.0" encoding="UTF-8"?
5 Page 5 | Supplementary Information

Supplementary Information

| Fig S2 Verification of the HSP23gene replacement by the respective resistance cassettes.  Sequence reads from each analysed strain (A-H) were aligned to the in silico-designed reference  DNA sequences consisting of the L. donovani genome (DNA) sequence of chromosome 34  (TriTrypDB-46\_LdonovaniBPK282A1\_Genome.fasta) with the expected insertion of antibiotic  resistance cassettes (NeoR = Neomycin; PuroR = Puromycin). The Y-axis represents the number  of reads and the X-axis shows the nucleotide position (bp) on chromosome 34. Grey shaded areas  denote complete lack of aligned reads. |

|  |
| Fig S3: Dendrogram of chromosomal ploidy profiles. Heat map showing somy levels across all  samples. The x-axis indicates individual chromosomes of L. donovani. Color scale indicates low  (blue) to high (yellow) ploidy. The dendrogram shows clustering of the strains based on similarity  of ploidy profiles and was generated by Pearson distance measurements. |
