# Supplementary material for: Casein kinase 1.2 over expression restores stress resistance to Leishmania donovani HSP23 null mutants
Source: Sci Rep. 2020 Sep 29;10:15969. doi: 10.1038/s41598-020-72724-x (PMC7525241; doi:10.1038/s41598-020-72724-x)
Supplement: Supplementary file 2 — Supplementary Information 2. [file 41598_2020_72724_MOESM2_ESM.epub › OPS/page-6.xhtml]

xml version="1.0" encoding="UTF-8"?
6 Page 6 | Supplementary Information

Supplementary Information

| Fig S4 Abundance of mRNA levels in transgenic over expression lines. mRNA was isolated  from transgenic over expression lines HSP23-/- [Mix]-LT and HSP23-/- [Mix]-HT, selected at 25°C  or 37°C, respectively. mRNA levels were determined for all genes of the six-gene cluster on  chromosome 35 (LdBPK\_351030-351080) and normalised to WT levels (set to 1). |
