# Supplementary material for: Casein kinase 1.2 over expression restores stress resistance to Leishmania donovani HSP23 null mutants
Source: Sci Rep. 2020 Sep 29;10:15969. doi: 10.1038/s41598-020-72724-x (PMC7525241; doi:10.1038/s41598-020-72724-x)
Supplement: Supplementary file 2 — Supplementary Information 2. [file 41598_2020_72724_MOESM2_ESM.epub › OPS/page-7.xhtml]

xml version="1.0" encoding="UTF-8"?
7 Page 7 | Supplementary Information

Supplementary Information

|  |
| Fig S5 Characterisation of CK1.2 over expression lines. (A) List of transgenic parasite cell  lines tested. NT sel = selected under nourseothricine; temp. sel. = temperature selection scheme.  (B-D) 1✕105 cells/ml were seeded into 7 ml of supplemented M199+ medium and grown for 4  days. Cell density on day 4 was then calculated as a percentage of wild-type (HSP23+/+) cell  density (set at 100%). Parasites were grown at 25˚C (B), 25°C and 2% EtOH (C), 25˚C and 200  nM H2O2 (D). Differences were tested for significance using the Kruskal–Wallis test: \*=p<0.05,  \*\*=p<0.01 (n=4) |
