# Supplementary material for: Casein kinase 1.2 over expression restores stress resistance to Leishmania donovani HSP23 null mutants
Source: Sci Rep. 2020 Sep 29;10:15969. doi: 10.1038/s41598-020-72724-x (PMC7525241; doi:10.1038/s41598-020-72724-x)
Supplement: Supplementary file 2 — Supplementary Information 2. [file 41598_2020_72724_MOESM2_ESM.epub › OPS/page-8.xhtml]

xml version="1.0" encoding="UTF-8"?
8 Page 8 | Supplementary Information

Supplementary Information

|  |
| Fig S6 Abundance of CK1.2 in HSP23-/- esc1/2 mutants under different temperatures. CK1.2  mRNA (n=2) (A) and protein (n=3) (B) abundance were determined for HSP23-/- esc1/2 mutants  that were selected first at HT (37°C) and were then transferred back to LT (25°C)(HT→LT).  mRNA and protein samples were taken 30 days after transfer to LT conditions. For Western blot  analysis, signal intensities were quantified using the Image J Software and were normalised to the  WT (set to 1). |

|  |
| Fig S7 Protein sequence alignments of HSP23 orthologs in Leishmania spp. and L. donovani  P23 protein. Putative CK1 phosphorylation sites (S/TXXS/T) are underlined and in bold font. |
