# Supplementary material for: Casein kinase 1.2 over expression restores stress resistance to Leishmania donovani HSP23 null mutants
Source: Sci Rep. 2020 Sep 29;10:15969. doi: 10.1038/s41598-020-72724-x (PMC7525241; doi:10.1038/s41598-020-72724-x)
Supplement: Supplementary file 2 — Supplementary Information 2. [file 41598_2020_72724_MOESM2_ESM.epub › OPS/page-9.xhtml]

xml version="1.0" encoding="UTF-8"?
9 Page 9 | Supplementary Information

Supplementary Information

|  |
| Fig S8 Schematic depiction plasmid used for protein expression in E.coli. pJC65 is derived  from pJC45 (Schlüter et al., 2000), with a newly designed multiple cloning site. |
