# Supplementary material for: Casein kinase 1.2 over expression restores stress resistance to Leishmania donovani HSP23 null mutants
Source: Sci Rep. 2020 Sep 29;10:15969. doi: 10.1038/s41598-020-72724-x (PMC7525241; doi:10.1038/s41598-020-72724-x)
Supplement: Supplementary file 2 — Supplementary Information 2. [file 41598_2020_72724_MOESM2_ESM.epub › OPS/page-13.xhtml]

xml version="1.0" encoding="UTF-8"?
13 Page 13 | Supplementary Information

Supplementary Information

￼
